# Single-Dose Creatine Reduces Sleep Deprivation-Induced Deterioration in Cognitive Performance

**DOI:** 10.3390/nu18081192

**Published:** 2026-04-10

**Authors:** Ali Gordji-Nejad, Andreas Matusch, Lea Hengstler, Simone Beer, Tina Kroll, Sabine Klein, David Elmenhorst, Andreas Bauer, Alexander Drzezga

**Affiliations:** 1 Institute of Neuroscience and Medicine (INM-2), Molecular Organization of the Brain, Forschungszentrum Jülich, 52425 Jülich, Germany; 2 Department of Nuclear Medicine, Faculty of Medicine and University Hospital Cologne, University of Cologne, 50937 Cologne, Germany; 3 German Center for Neurodegenerative Diseases (DZNE), 53127 Bonn, Germany

**Keywords:** creatine, sleep deprivation, cognitive performance

## Abstract

**Background/Objectives:** Creatine is a supplement that, beyond its physiological effects, has been shown to have positive effects on cognitive abilities. In our previous study, we showed that a single dose of 0.35 g/kg creatine induces changes in brain metabolism during sleep deprivation and reduces deterioration in cognitive performance. The present study investigates whether supplementation of a lower dose is associated with cognitive effects during sleep deprivation, focusing exclusively on cognitive performance outcomes. **Methods**: Twenty-nine healthy subjects performed cognitive tests at the evening baseline and 3, 5.5, and 7.5 h after receiving a single dose of creatine monohydrate (0.2 g/kg) or a placebo during a total of 21 h of sleep deprivation (SD). **Results:** The results show a mitigating effect of creatine on sleep deprivation-induced deterioration in logical and numerical tasks, language-related processing speed, and the Psychomotor Vigilance Test (PVT). Compared to males, females benefit more in logic, PVT and processing speed in language and logic tasks. **Conclusions:** Our results show that a dose of 0.2 g/kg creatine is associated with a reduced deterioration in cognitive performance during sleep deprivation. Although the effect is less pronounced than with a high dose of 0.35 g/kg, there is still an improvement of up to 12%.

## 1. Introduction

Creatine is a supplement primarily used by the sports community to improve physical performance. However, recent research findings have revealed that its potential applications extend far beyond the field of fitness. The clinical relevance and therapeutic effects of creatine [1,2,3,4] have been demonstrated in cases of traumatic brain injury (TBI) [2,5], neurodegenerative diseases [3], tumor-suppression [6] and depression [4,7]. Furthermore, creatine has been shown to positively affect cognition [8,9], prevent age-related muscle loss and increase bone density [10]. It can influence neurocognitive and metabolic changes during menopause in women [11,12,13] and offer benefits in cases of sleep deprivation (SD), fatigue, or cognitive impairment [8,14,15,16,17]. There is growing evidence indicating that creatine influences neuronal excitability and neurotransmission. It modulates inhibitory neurotransmissions by interacting with several receptor systems, including adenosine N-methyl-D-aspartate (NMDA) and γ-aminobutyric acid type A (GABA_A_), and appears to fine-tune the pathways involved in mood regulation by influencing dopamine (D_1_ and D_2_), serotonin (5-HT_1A_), α1-adrenoceptors and adenosine (A1 and A2a) receptors [2,18,19,20,21]. In addition, creatine exhibits neuroprotective properties by revealing antioxidant activity via the sequestration of reactive oxygen species (ROS) and reduction in glutamate-mediated and amyloid-β (Aβ) toxicity [22,23]. The factors above play a major role in depression, Parkinson’s and Alzheimer’s diseases, synaptic plasticity, learning memory and sleep–wake regulation.

However, in healthy individuals or at rest, creatine appears to have little or no effect on brain creatine levels [24,25]. In all the above cases, a cellular stress state appears to be a decisive condition for an increased uptake. Due to its low absorption rate in the central nervous system (CNS), a diet of at least one week of oral creatine supplementation is needed to induce neurometabolic changes or positive cognitive effects [9,26,27,28]. This poses a particular challenge in cases of sleep deprivation, where short-term effects are crucial.

In our previous study, we demonstrated that a high single dose of 0.35 g/kg creatine induces metabolic changes and attenuates cognitive impairment during SD [14]. Here, cellular stress induced by sleep deprivation, combined with high extracellular availability of creatine resulting from a high dose, appeared to provide the conditions for a higher intracellular uptake. If both factors are responsible for increased creatine absorption, the question arises of whether a customary dose is sufficient to achieve the same effect, as this minimizes side effects and improves the safety of consumers. Since creatine also appears to have positive effects on stress states in the brain and might have potential benefits in neurodegenerative diseases, research on dose effects is also necessary [29]. In this study, we used the same design as previously published [14,30], but using a lower creatine dose and focusing on cognitive performance. Changes in cognitive performance were investigated over an eight-hour period following a single acute dose of creatine (0.2 g/kg) vs. placebo during the first two-thirds of a sleepless night. Cognitive tasks were performed at four measurement time points.

## 2. Materials and Methods

### 2.1. Study Participants

Twenty-nine healthy subjects (17 females, 9 vegetarians, aged 27 ± 6 years, range 20–40, 25 right-handed) participated. None of the subjects reported sleep disorders, psychiatric or neurological conditions, or alcohol or drug abuse. None of them smoked or took medication. Consumption of caffeine and alcohol on an occasional basis was discontinued at least 48 h before the measurement nights. Subjects were required to sleep for at least seven hours every night during the two weeks prior to and between measurement days, to go to bed by 11 pm on the preceding night, and to wake up at 7 am They were requested to record all sleep and wake times. The study was conducted in accordance with the Declaration of Helsinki and was approved by the Ethics Committee of Ärztekammer Nordrhein (North Rhine State Chamber of Physicians, Düsseldorf, Germany.). All subjects gave their consent to participate in the study. The study was retrospectively registered in the German Clinical Trials Register (DRKS; registration number: DRKS00039845) as it involved healthy volunteers and a nutritional intervention, which the authors initially did not categorize as a clinical trial requiring prospective registration.

### 2.2. Experimental Procedure

**Figure 1:**
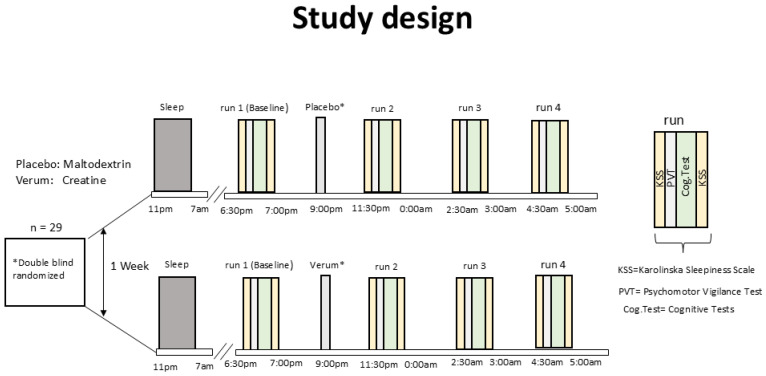
**Study design**: In a double-blind, randomized, cross-over design (*), subjects were administered creatine at 9:00 pm in one session and placebo in another, spaced at 7 days in random order. Cognitive parameters were acquired in four runs: baseline starting at 6:30 pm, and the others at 11:30 pm, 2:30 am, and 4:30 am. Each session took 25 min and comprised two fatigue score questionnaires, Psychomotor Vigilance Tests (PVTs), and cognitive tasks (Cog.Test).

This study was a double-blind, randomized, prospective trial with a balanced crossover design. Verum (Creavitalis, creatinemonohydrate micronized, AlzChem, Trostberg, Germany) and placebo (Resistant Dextrin, edubily GmbH, Wallerfangen, Germany) were prepared, blinded, and arranged in pair-wise randomized, balanced order by the Rosen Pharmacy (Cologne, Germany). Every subject was measured on two nights with a 7-day interval in between (Figure 1).

Subjects were not allowed to sleep throughout both sessions and were permanently supervised and monitored. No cognitively stressful activities were permitted, while the consumption of water and nonmeat snacks was allowed. On one night, 0.2 g/kg creatine was administered, on the other, a 0.2 g/kg placebo.

Following the baseline measurement, which began at 6:33 pm (±31 min), subjects received either 0.2 g/kg creatine or 0.2 g/kg placebo orally at 9:00 pm (±29 min). Further measurements were taken at 11:33 pm (±12 min), 2:29 am (±28 min), and 4:29 am (±27 min), referred to hereafter as 12 am, 2 am, and 4 am. Each measurement included cognitive tasks lasting 22 min and 48 s (±5 min).

#### 2.2.1. Cognitive Scores and Tasks

The positive and negative affect schedule, PANAS [31], was acquired initially once per measurement night. At the beginning and end of each session, participants completed the Karolinska Sleepiness Scale (KSS; ranging from 1 to 10) and a fatigue scale (FAT; ranging from 1 to 20). The FAT scale consisted of an inverted 10-item version of the Samn–Perelli Fatigue Scale [32,33].

The test battery consisted of a Psychomotor Vigilance Test (PVT, 8 min), Word Memory Test (WMT, 1 min:54 s ± 42 s), forward digit span (span, 27 s ± 11 s), spatial N-back (2-back) (1 min:3 s ± 6 s) and multiple-choice tasks in language (3 min:37 s ± 74 s), logic (3 min:59 s ± 66 s) and numeric ability (3 min:33 s ± 93 s). The processing time of each task was recorded. Before beginning the study, each subject completed a training run of all tasks.

##### PVT (Psychomotor Vigilance Test)

PC-PVT 2.0, a software program analogous to PVT-192 [34] freely available at https://pcpvt.bhsai.org, (1 August 2025), was used to measure the reaction time. The task was performed on a laptop in a dark environment and lasted 8 min. Subjects had to press the mouse button as soon as a counter started on a black background.

##### Memory Tasks

The Word Memory Test (WMT) and the SPAN task were implemented in Microsoft Excel using Visual Basic for Applications (VBA). The WMT, adapted from [35,36], comprised 22 pairs of German nouns (e.g., “climate”–“storm”). To minimize primacy and recency effects, four additional filler pairs were presented at the beginning and at the end of the task. Each word pair was shown for five seconds. During the recall phase, participants were required to enter the second word of each pair immediately after the first word was displayed, without any time restriction. The SPAN task consisted of 12 randomly generated single-digit numbers, each presented for five seconds. Subsequently, participants were required to recall the digits in their original order, without any time constraints. For both tasks, eight different lists were created for eight sessions. The sequence of lists, as well as the order of word pairs and numbers within each list, was randomized across participants. The spatial triple N-back (2-back) task was administered using Brain Workshop software [37] (version 4.8.4). It comprised two consecutive 63 s blocks, each containing 21 trials of three seconds. Squares were displayed sequentially within a 3 × 3 grid, and participants were instructed to press a button whenever the current square matched the one presented two trials earlier.

##### Cognitive Multiple-Choice Tests

The categories covered were language (21 tasks), logic (17 tasks), and numeric ability (9 tasks), chosen from the IQ-Test training [38]. Language sub-categories comprised finding analogies (5 tasks), arranging 5 letters to one word (7 tasks), finding words with common generic terms (4 tasks) and those not matching a list (5 tasks). Logical tasks were the completion of figure patterns (8 tasks) and mental rotation, such as rotating and flipping figures (4 tasks), mapping and folding figures (3 tasks), and turning and tilting of dice (2 tasks). Numeric tasks comprised completing number sequences by finding the pattern (4 tasks) and addition of numbers (5 tasks). A total of 8 different batteries were prepared for 8 runs and divided into series A and B (each containing 4 batteries) for the two measurement nights. The order of both series was randomized and balanced over subjects.

### 2.3. Statistics and Evaluation

With a desired statistical power of 0.80, the expected mean difference from the previous study [14] was adjusted to the conditions of the lower dosage used in the present study, resulting in an effect size of 0.72 (mean difference = 1.2, correlation = 0.8, standard deviation = 2.7). The G*Power calculator (Version 3.1.9.7, HHU-Düsseldorf) yields a required sample size of *n* = 17 for the intra-individual comparison of two sessions at a significance level of 0.05. Primary longitudinal data were analyzed using a Linear Mixed Model (LMM) implemented in Python (Version 3.11.9, statsmodels 0.14.5) to account for the hierarchical structure of the repeated measurements. Data obtained during the sleep deprivation protocol was nested within participants, and subject ID was included as a grouping factor. Time was modeled as a continuous variable representing hours since baseline assessment (0, 6, 8, 10). For all models, both a random intercept and a random slope for time were specified at the participant level. Vegetarian status and sex were included as fixed-effect covariates to control for potential baseline and trajectory differences associated with dietary pattern and biological sex.

To quantify the net effect of each condition, intra-individual changes relative to baseline (6 pm) were calculated and pooled across three post-supplementation intervals (12 am, 2 am, and 4 am) by using two-tailed paired-sample tests. The net protective effect of creatine against sleep deprivation-induced cognitive decline was then calculated using a difference-in-differences (DiD) approach by subtracting the baseline-corrected change observed under placebo from the corresponding change under creatine (Supplementary Materials). For multiple testing control, significance thresholds were adjusted according to the Bonferroni approach. As measurements were carried out in eight conditions (*k* = 8, 2 sessions, each with 4 runs), the α thresholds were adjusted to α = 0.05/8 =0.0063. Correlations in changes in fatigue and cognitive scores were analyzed by calculating the Pearson correlation coefficient (r).

## 3. Results

All 29 participants successfully completed the study. Creatine supplementation was well tolerated, with no reports of gastrointestinal distress or other adverse physical symptoms. Continuous monitoring was employed to verify sustained wakefulness throughout the sessions. A query before the study started regarding the requested sleep time schedule within the previous two weeks did not result in any significant deviations. The SD was effective and had a significant impact on the specified parameters. With four sessions for each subject per night, a total of 1624 tasks were evaluated in 232 sessions for all participants. Only one SPAN score at the time point 12 am from one subject was not recorded, and an extreme PVT score from one session of another subject was removed.

**Figure 2:**
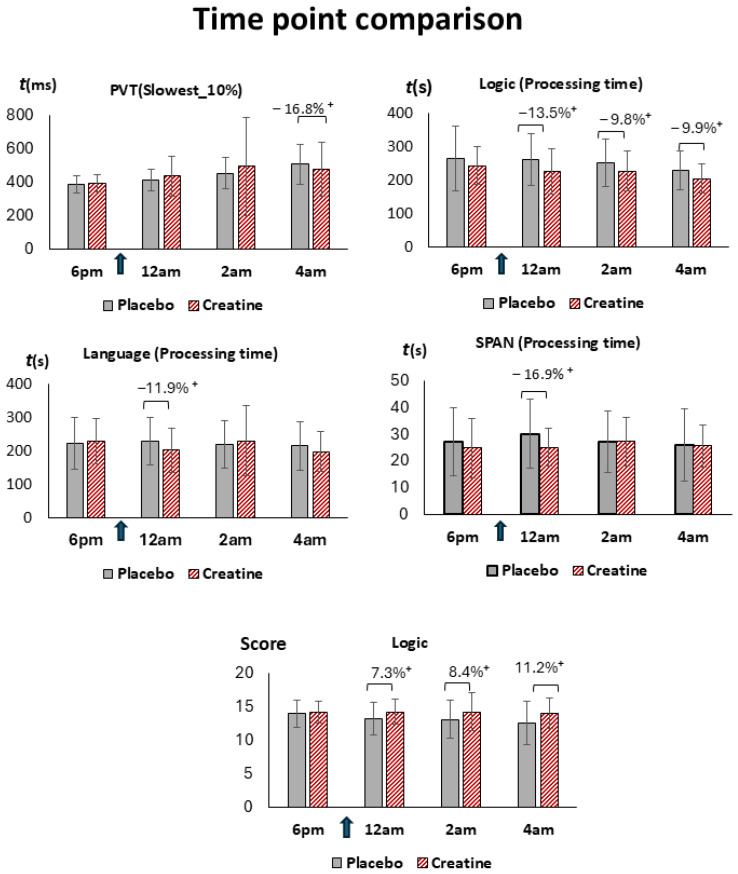
**Score and processing time** of selected cognitive parameters during sleep deprivation under placebo (gray) and creatine (red, hatched). Shown are results in Psychomotor Vigilance Test (PVT, reaction time RT), logic, language and forward digit span (SPAN) tasks. Arrows indicate administration of creatine or placebo at 9:00 pm. (+) denotes significant difference (^+^ *p*_27_ ≤ 0.05) of creatine compared to placebo. Bars represent standard errors (SE).

**Figure 3:**
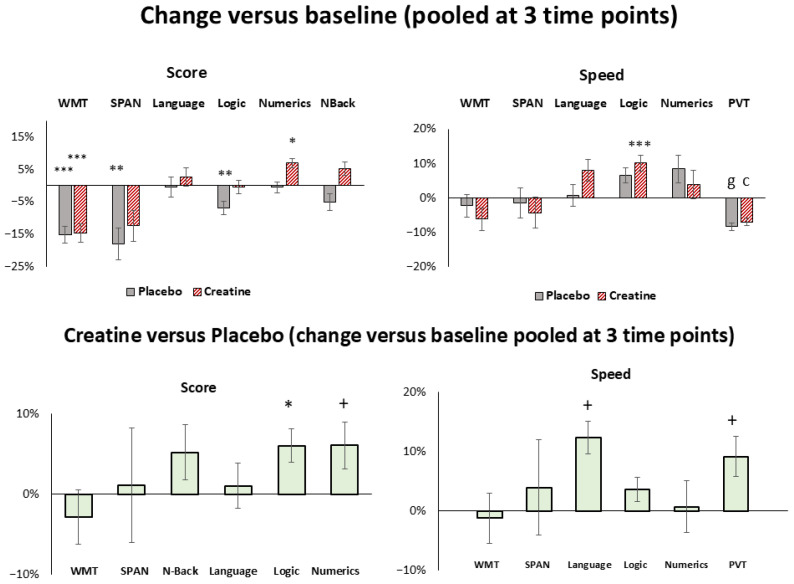
**Baseline (6 pm)-related percentage changes in cognitive performance** during sleep deprivation under placebo (gray), creatine (red, hatched) and creatine versus placebo, when pooled at all 3 time points (12 am, 2 am, 4 am). Improvements in task performance are shown as positive values and deteriorations as negative values. Shown are language, logic, numeric ability, forward digit span (SPAN), word memory tasks (WMTs), N-Back and Psychomotor Vigilance Test (PVT, reaction time (RT), reaction time dispersion (RTD) for creatine vs. placebo). Significance levels are presented by ^+^ *p*_85_ ≤ 0.05, * *p*_85_ ≤ 0.005, ** *p*_85_ ≤ 0.0005, *** *p*_85_ ≤ 0.00005, ^c^ *p*_85_ ≤ 5.0 × 10^−8^ and ^g^ *p*_85_ ≤ 5.0 × 10^−12^. Bars represent standard errors (SE).

**Figure 4:**
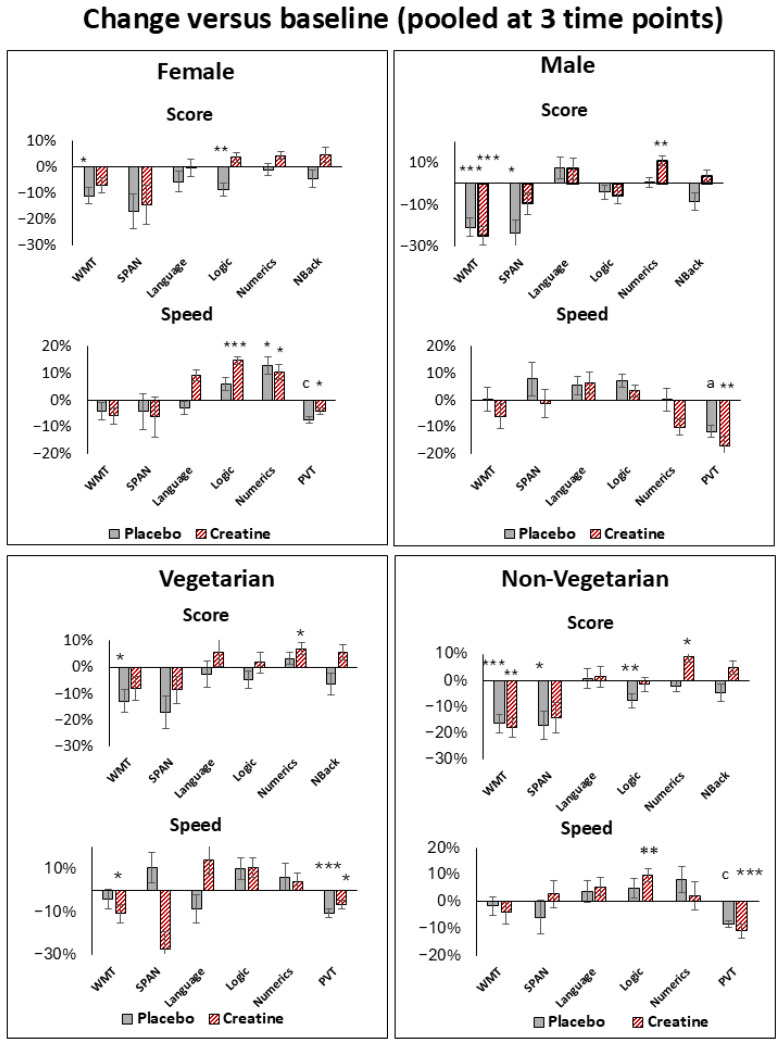
**Baseline (6 pm)-related percentage changes in cognitive performance** for female, male, vegetarian and non-vegetarian subjects during sleep deprivation under creatine versus placebo when pooled at all 3 time points (12 am, 2 am, 4 am). Improvements in task performance are shown as positive values and deteriorations as negative values. Shown are language, logic, numeric ability, forward digit span (SPAN), Word memory tasks (WMTs), N-Back and Psychomotor Vigilance Tests (PVTs, reaction time). (RT). Significance levels are presented by * *p*_85_ ≤ 0.005, ** *p*_85_ ≤ 0.0005, *** *p*_85_ ≤ 0.00005, ^a^ *p*_85_ ≤ 5.0 × 10^−6^ and ^c^ *p*_85_ ≤ 5.0 × 10^−8^. Bars represent standard errors (SE).

**Figure 5:**
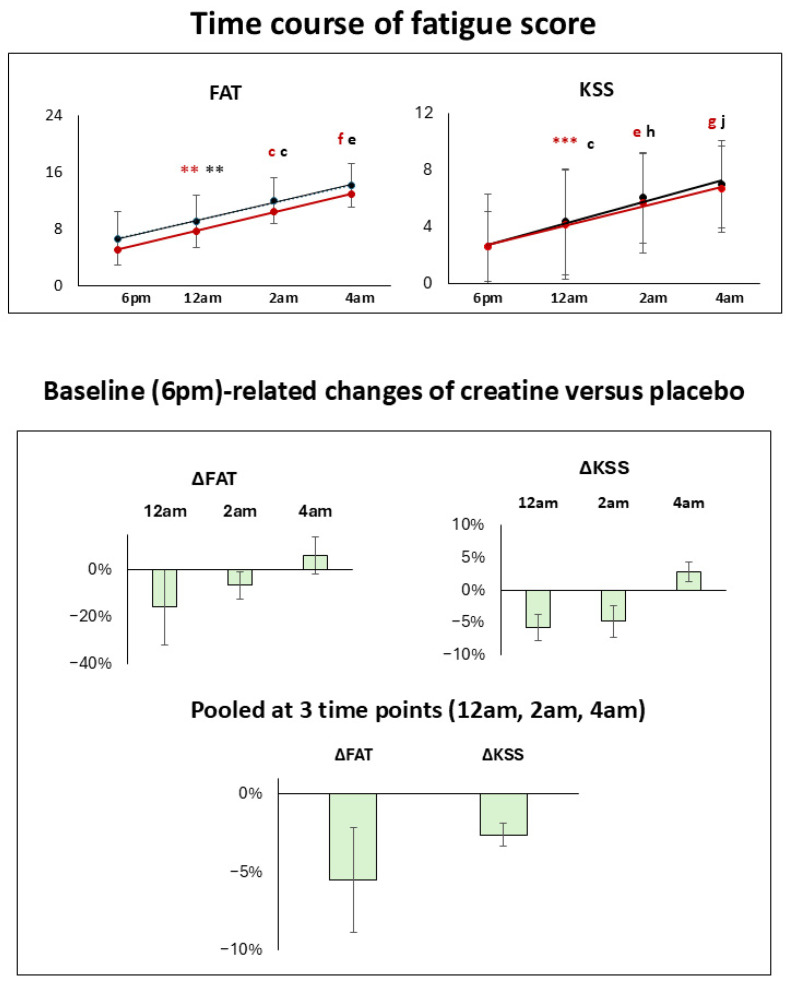
(**Top**): Time course of fatigue scale (FAT) and Karolinska Sleepiness Scale (KSS) for placebo (black) and creatine (red) at all time points. Significance levels represent the differences from the baseline level. (**Below**): Baseline (6 pm)-related percentage changes of creatine versus placebo at each time point and when pooled at all 3 time points. Positive values denote an increased fatigue state and negative values a decreased fatigue state. Significance level is presented by ** *p*_85_ ≤ 0.0005, *** *p*_85_ ≤ 0.00005, ^c^ *p* ≤ 5.0 × 10^−8^, ^e–h^ *p* ≤ 5.0 × 10^−10^ to 5.0 × 10^−13^ in decadal steps and ^j^ *p* ≤ 5.0 × 10^−15^.

**Figure 6:**
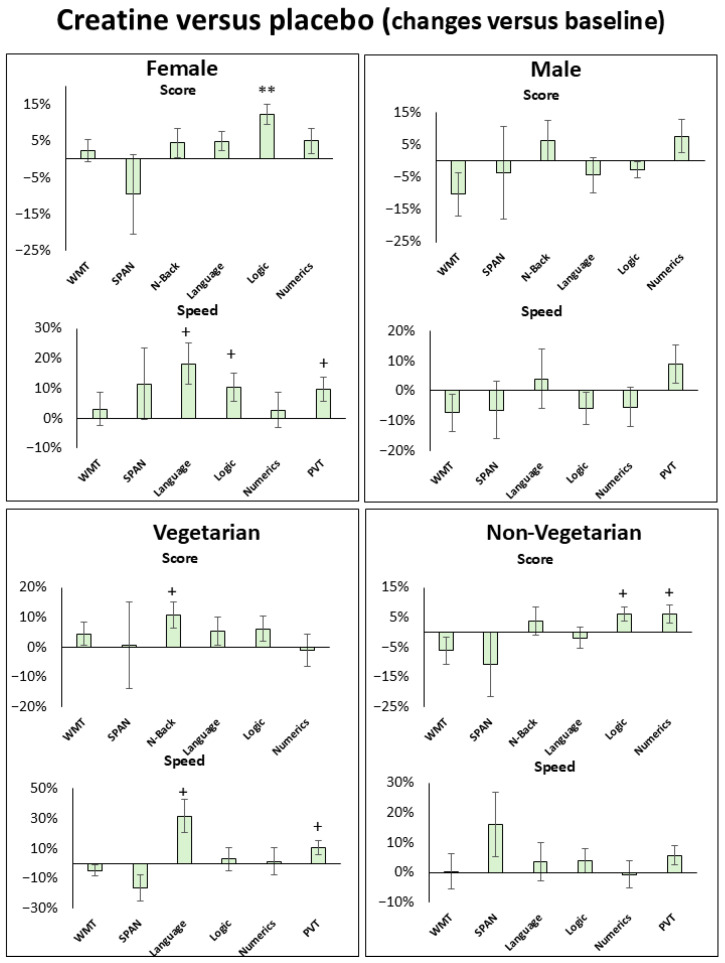
**Baseline (6 pm)-related percentage changes in cognitive performance** for female, male, vegetarian and non-vegetarian subjects during sleep deprivation under creatine versus placebo when pooled at all 3 time points (12 am, 2 am, 4 am). Improvements in task performance are shown as positive values and deteriorations as negative values. Shown are language, logic, numeric ability, forward digit span (SPAN), word memory tasks (WMTs), N-Back and Psychomotor Vigilance Tests (PVTs, reaction time dispersion (RTD), slowest 10% reaction time for vegetarians). Significance levels are presented by ^+^ *p*_85_ ≤ 0.05 and ** *p*_85_ ≤ 0.0005. Bars represent standard errors (SE).

The results of the cognitive tasks at selected time points and fatigue scales and their mean changes from baseline in both conditions for all subjects, females, males, vegetarians, and non-vegetarians are given in Table 1, Table 2, Tables S1–S4 and S9–S12, and Figure 2, Figure 3, Figure 4, Figure 5 and Figures S1–S3. Baseline-related changes in cognitive parameters of creatine versus placebo are given in Table 2, Table 3 and Tables S5–S12, and Figure 3, Figure 6, Figures S1 and S4. In the figures, improvements in task performance are shown as positive changes and deteriorations as negative changes. Fatigue scales and their mean within-subject changes versus baseline in both conditions and of creatine versus placebo are presented in Table 1, Table 3 and Tables S1–S8, and Figure 5. Correlations of cognitive response and fatigue scale are shown in Table 4, Tables S13 and S14. PANAS did not differ significantly between sessions, with positive and negative subscales amounting to 3.12 ± 0.7 and 1.3 ± 0.7 in the placebo and 3.3 ± 0.7 and 1.2 ± 0.2 in the creatine session. Except for the pooled creatine-versus-placebo comparison, results not surviving Bonferroni correction are presented in the Supplementary Materials.

### 3.1. Linear Mixed Model (LMM)

Baseline (6 pm)-related time interaction effect under placebo revealed in logic (*p* = 0.003), PVT (Mean_RT, *p* < 0.001; Median_RT, *p* < 0.001, RTD, *p* < 0.001), WMT (*p* < 0.001), SPAN (*p* = 0.001), KSS (*p* < 0.001) and FAT (*p* < 0.001) scales. Creatine treatment interaction effect revealed in logic (*p* = 0.044) and PVT (Mean_RTD, *p* = 0.046). For logic, creatine reduces the slope of decline significantly (*p* = 0.043) from −0.126 per hour under placebo to −0.015 per hour.

### 3.2. Difference-in-Differences (DiD)

#### 3.2.1. Cognitive Response to SD

Under placebo, KSS and FAT scores progressively increased in all subjects versus baseline, indicating an increased fatigue state. At the final time point (4 am), values had increased by 173 ± 5% (*p*_27_ = 1.5 × 10^−15^, *t*_27_ = 16.4) and 115 ± 18% (*p*_27_ = 2.6 × 10^−10^, *t*_27_ = 9.7), respectively (Table 1, Figure 5).

Deteriorations in memory tasks versus baseline appeared significantly by a negative percentage change in WMT at 2 am (−17.2 ± 4.6%, *p*_27_ = 0.001, *t*_27_ = −3.9); 4 am (−23.9 ± 6.4%, *p*_27_ = 0.0001, *t*_27_ = −4.5) and in SPAN (−18.0 ± 4.8%, *p*_85_ = 0.0003, *t*_85_ = −3.7), WMT (−15.2 ± 2.7%, *p*_85_ = 1.0 × 10^−7^, *t*_85_ = −5.8) and logic tasks (−6.8 ± 1.7%, *p*_85_ = 0.0001, *t*_85_ = −4.1) when pooled at all three time points (Table 1, Figure 3). Further deteriorations occurred in PVT by an increased reaction time at 2 am (8.3 ± 2.0%, *p*_27_ = 5.0 × 10^−5^, *t*_27_ = 4.9); 4 am (13.1 ± 2.0%, *p*_27_ = 3.1 × 10^−7^, *t*_27_ = 6.9) and when pooled at all three time points (8.4 ± 1.0%, *p*_85_ = 4.4 × 10^−12^, *t*_85_ = 8.1) (Table 2, Figure 3, negative entry as reduced speed in Figure 3). Significant changes in other PVT parameters are presented in Table 2. Significant positive correlations were found in KSS and PVT when pooled at all three time points (0.43, *p*_85_ = 0.00003, *t*_85_ = 4.4). This indicates that an increased fatigue state was associated with a deterioration in reaction time performance. Further changes at each time point for all subjects, females, males, vegetarians and non-vegetarians are presented in Table 1 and Table 2 and in Supplementary Materials (Tables S1–S4, Figures S1–S3). Although most of the single-time-point changes do not withstand the Bonferroni correction, there is a gradual deterioration in the tasks towards later time points, more pronounced in males and non-vegetarians than in females and vegetarians.

#### 3.2.2. Cognitive Response to SD After Creatine Administration

Following creatine administration, an increased fatigue state was revealed by a progressive increase in KSS and FAT scores relative to baseline. At the final time point (4 am), these values increased by 155 ± 6% (*p*_27_ = 2.8 × 10^−12^, *t*_27_ = 11.9) and 148 ± 12% (*p*_27_ = 5.7 × 10^−12^, *t*_27_ = 11.6) (Table 1, Figure 5), respectively.

Significant negative percentage changes, indicating deteriorations in memory tasks relative to baseline, were observed in the WMT at 2 am (−17.6 ± 4.9%, *p*_27_ = 0.001, *t*_27_ = −3.9); 4 am (−20.5 ± 6.3%, *p*_27_ = 0.001, *t*_27_ = −3.9) and when pooled at all three time points (−14.7 ± 2.8%, *p*_85_ = 1.2 × 10^−6^, *t*_85_ = −5.2) (Table 1, Figure 3).

Further deteriorations occurred in PVT by an increased reaction time at 2 am (7.4 ± 1.8%, *p*_27_ = 0.0005, *t*_27_ = 4.0); 4 am (9.4 ± 2.0%, *p*_27_ = 0.0005, *t*_27_ = 4.0) and when pooled at all three time points (7.0 ± 1.1%, *p*_85_ = 3.6 × 10^−8^, *t*_85_ = 6.1) (Table 2, Figure 3, negative entry as reduced speed in Figure 3). Changes in females, males, vegetarians and non-vegetarians are presented in Supplementary Materials (Tables S1–S4, Figure 4).

Improvements occurred in numeric task (6.9 ± 2.1%, *p*_85_ = 0.001, *t*_85_ = 3.4) and in speed in processing time in language (8.0 ± 3.1%, *p*_85_ = 0.01, *t*_85_ = 2.6) and logic (10.1 ± 2.2%, *p*_85_ = 2.0 × 10^−5^, *t*_85_ = 5.5) tasks when pooled at all three time points. Further improvements occurred in speed in processing time in logic tasks at 4 am (16.3 ± 4.4%, *p*_27_ = 0.001, *t*_27_ = 3.7). Changes at each time point for all subjects, females, males, vegetarians and non-vegetarians are presented in Table 1 and Table 2 and in Supplementary Materials (Tables S1–S4, Figures S1–S3). While most changes did not remain significant after Bonferroni correction, improvements were more prominent at earlier rather than later time points in males, vegetarians, and non-vegetarians.

#### 3.2.3. Cognitive Response with Creatine Versus Placebo

Improvements in logic (6.1 ± 2.1%, *p*_85_ = 0.005, *t*_85_ = 2.9), numeric (6.2 ± 2.9%, *p*_85_ = 0.04, *t*_85_ = 2.1), speed in processing time in language (12.3 ± 5.8%, *p*_85_ = 0.04, *t*_85_ = 2.1), and reaction time dispersion (RTD) in PVT (9.2 ± 3.4%, *p*_85_ = 0.02, *t*_85_ = 2.4) occurred when pooled at all three time points (Table 3, Figure 3). Females showed improvements in logic (12.3 ± 2.8%, *p*_49_ = 0.0001, *t*_49_ = 4.4), speed in processing time in language (18.2 ± 6.9%, *p*_49_ = 0.01, *t*_49_ = 2.6), logic (10.3 ± 4.7%, *p*_49_ = 0.03, *t*_49_ = 2.2) and PVT (RTD = 9.6 ± 1.4%, *p*_49_ = 0.04, *t*_49_ = 2.1, Mean RT = 4.3 ± 1.4%, *p*_49_ = 0.01, *t*_49_ = 2.6, Median RT = 3.9 ± 1.3%, *p*_49_ = 0.02, *t*_49_ = 2.4, Speed = 3.2 ± 1.2%, *p*_49_ = 0.02, *t*_49_ = 2.4) (Tables S5 and S9, Figure 6). Vegetarians revealed improvements in N-back (10.8 ± 4.4%, *p*_25_ = 0.02, *t*_25_ = 2.4), speed in processing time in language tasks (31.6 ± 11.1%, *p*_25_ = 0.01, *t*_25_ = 2.8) and PVT (Slowest 10% Mean RT = 10.6 ± 4.7%, *p*_49_ = 0.04, *t*_49_ = 2.2); and non-vegetarians improved in logic (6.0 ± 2.3%, *p*_58_ = 0.01, *t*_58_ = 2.6) and numeric (6.1 ± 2.9%, *p*_58_ = 0.04, *t*_58_ = 2.1) tasks (Tables S7 and S8, Figure 6). Further changes notwithstanding, the Bonferroni correction revealed improvements in scores among females and vegetarians, as well as in processing speed among non-vegetarians, showing no specific temporal trend (Supplementary Materials Tables S5–S8, Figure S4).

## 4. Discussion

This randomized, controlled, double-blind, crossover study investigated the cognitive response to a single oral dose of creatine (0.2 g/kg) compared to baseline and placebo during sleep deprivation. The SD-induced deterioration in cognitive performance was mitigated across several tasks. These results replicate and confirm the acute effects of a single creatine dose on sleep deprivation.

Creatine plays a pivotal role in maintaining cellular energy homeostasis by enabling creatine kinase (CK)-mediated regeneration of ATP, the brain’s primary energy currency [39,40,41]. Prolonged metabolic demands, such as cognitive activity during sleep deprivation, deplete cellular energy reserves and lead to impaired performance. Maintaining optimal creatine levels through high-dose administration can accelerate resynthesis and replenish depleted cellular reserves, thereby protecting cognitive function against metabolic exhaustion.

A major limitation of this approach is the typically restricted cellular uptake of creatine. However, sleep deprivation appears to trigger stress-related mechanisms that enhance intracellular creatine influx [42,43,44,45,46,47,48,49]. As a result, the sleep deprivation-induced decline in cognitive performance can be temporarily attenuated, as demonstrated in our previous study [14].

These findings raised the question of whether the sleep deprivation-induced cellular stress state alone drives the increased intracellular creatine uptake, or whether the high extracellular availability of creatine provided by the large dose also plays a critical role. Therefore, using the same study design, we investigated whether a lower dose of 0.2 g/kg would produce cognitive effects too. Due to the absence of metabolic brain measurements and statistical comparisons with our previous study, direct comparisons between studies are not possible and any interpretation of differences should be made with caution.

Our results indicate that a beneficial effect is still present at the lower dose, although less pronounced, suggesting that both factors—the cellular stress state and sufficient extracellular creatine availability—are essential for the observed response.

In contrast to the higher dose used in our previous study, the strongest effects were not observed in short-term memory tasks or processing speed, but rather in PVT and working-memory domains, particularly in logic tasks. Specifically, improvements compared to placebo were characterized by an increase in task scores and enhanced speed performance relative to baseline (Figure 3). Nevertheless, exploratory paired *t*-test comparisons at individual measurement time points (Figure 2) suggested a trend toward improved processing speed. Although these effects did not remain statistically significant after correction for multiple comparisons, their direction was consistent with the pattern observed at the higher dose.

Our results emphasize that the effects of creatine on short-term memory appear to be dose-dependent. The observed pattern resembles peak performance during high-intensity physical exercise, where creatine exerts its strongest effects during short, intense bursts of activity. In such conditions, creatine supports an anaerobic alactic energy state by buffering ATP levels and limiting excessive lactate accumulation at maximal exertion [50,51].

Previous research indicates a non-linear dose–response relationship for creatine, whereby higher doses during the loading phase lead to faster tissue saturation and, consequently, greater peak performance. Pharmacokinetic studies focusing on plasma and muscle have shown that creatine is rapidly absorbed after ingestion, reaching peak blood concentrations (Cmax) within approximately 2–3 h [52,53]. Moreover, both serum creatine levels and peak time concentration increase linearly with dosage (0, 10, 20, and 30 g), with maximal levels typically observed after about three hours. At this time point, substantial differences in serum concentrations can be observed between high (20 g; c(Cr) ≈ 2000 µmol/L) and low (10 g; c(Cr) ≈ 600 µmol/L) doses. In our study, the mean administered creatine dose was 14.1 ± 3 g. Given the reduced influx into the neuronal intracellular space, significantly lower absorption in neuronal cells than in muscles can be expected. Therefore, it can be concluded that the effective cellular uptake and the associated effect on cognitive performance of the reduced dose may reach their limit.

While the customary daily dose ranges from ~0.03 g/kg to 0.1 g/kg [54,55], recent meta-analyses have shown that a creatine supplementation dosing regimen of 0.2 g/kg is safe [56]. Although our statistical analysis showed improvement in several categories, an intra-individual comparison of creatine versus placebo (Figure 3) showed a maximum improvement ranging from 6% to 12%. If these results indicate the limits of creatine are reached in the used dose of 0.2 g/kg, a focus for future studies on use by normal or professional consumers could be to determine whether creatine in modified form or mixed with additional components could increase cellular uptake [2,57,58].

Further notable are the differences observed when comparing the sexes. Specifically, sex-stratified analyses suggested a more pronounced response in female participants. Although the primary mixed-effects model did not reach the threshold for a significant temporal interaction, DiD analyses revealed a significant attenuation of sleep deprivation-induced performance decline following creatine supplementation (Figure 6). This protective pattern showed cross-domain consistency, spanning logic, and processing speed in language, logic and PVT, and was mirrored by an increase in task scores and performance speed relative to baseline. These findings suggest that females may exhibit greater sensitivity to the bioenergetic support provided by creatine during acute sleep deprivation, underscoring the importance of considering sex as a critical biological variable in studies of metabolic cognitive enhancement.

Females appeared to be less affected by a decline in cognitive performance than males, particularly regarding processing speed and reaction time (PVT) (Figure 6). This outcome is supported by a lower dependence of performance in reaction time (PVT) on fatigue (KSS), which is known to be a sleep deprivation-sensitive task. A significant correlation occurred here in males, but not in females (Table S13). The resilience of females to fatigue and sleep deprivation, particularly with regard to vigilance tasks, is consistent with that observed in other studies [59,60,61]. An fMRI study showed higher regional homogeneity (ReHo) in neuronal activity in the right frontal lobe in females compared to males after sleep deprivation, a region responsible for attention, alertness and executive control [62]. Although females appear to be more resilient, our results suggest that they also respond more strongly to creatine than males, particularly in logic tasks and processing speed (Figure 6).

From a metabolic point of view, the resilience to sleep-deprived cognitive decline in females is also consistent with the results of our previous study [30]. Male participants showed a higher hemispheric asymmetry in metabolic energy consumption than females. The affected right hemisphere is relevant for attention-related tasks. Creatine compensates for this asymmetry. It is therefore remarkable that creatine nevertheless has a greater effect on females. Due to a reported lower regional creatine level in the brain of females [63], a higher demand can be assumed, as is also the case in vegetarians. Research demonstrates that creatine levels and synthesis in the female brain are estrogen-dependent and exhibit fluctuations throughout the menstrual cycle and lifespan. Specifically, the reductions observed during the luteal phase and menopause [11,64,65] result in periodic instability in cerebral energy availability. This regionally concerns the prefrontal cortex, a region responsible for decision-making and logical tasks. The strong effect of creatine on females may also stem from genetic factors. Creatine uptake is highly dependent on the SLC6A8 transporter gene, which is located on the X chromosome. Due to X inactivation or genetic variation, females may have different transporter levels, which can lead to higher or more stable transporter expression [66,67].

Furthermore, the DiD analyses revealed that both the vegetarian and non-vegetarian groups showed significant improvements, although the comparison revealed fewer differences than that between the genders (Figure 6). 

Nevertheless, there are similarities between females and vegetarians. As with females, vegetarians also showed improvements in processing speed. Assuming a higher demand in both groups due to reduced creatine levels, this outcome supports the significant impact on peak performance, as is the case with athletes [68]. However, several limitations should be considered when interpreting these findings. Although dietary intake was restricted to meat-free meals, total caloric intake was not strictly controlled, which may have influenced performance in some of the included tasks [69]. In addition, despite an increased sample size compared to our previous study, the overall number of participants (*n* = 29) remains relatively small. Furthermore, no direct measures of brain creatine levels were obtained, and no statistical correlation analyses were conducted, limiting quantitative interpretation. Finally, the sample consisted exclusively of young adults, and the findings may therefore not be generalizable to older populations.

Nevertheless, if these results are confirmed in older adults by further studies, they will play a key role in developing personalized supplementation strategies. Thus, women, vegetarians, and older adults—groups with a tendency toward lower creatine stores—would benefit most from supplementation during sleep deprivation. Particularly for athletes and coaches, a dose-dependent strategy is recommended to mitigate the cognitive costs of sleep deprivation. While a moderate dose provides modest improvements, a higher dose appears necessary to achieve peak performance, particularly in tasks requiring high processing speed. These findings provide a basis for customizing dosages to specific populations and ensuring the safe use of creatine.

## 5. Conclusions

Our results show that administration of a dose of 0.2 g/kg creatine reduces, to a modest extent, the deterioration in cognitive performance during sleep deprivation. Although the effect is weak, an improvement of up to 12% is still observed. The results show mitigating effects on performance deterioration, which were most pronounced in logic, numerical ability, and processing speed in language tasks, as well as in the Psychomotor Vigilance Test (PVT). Compared to males, females benefited more in logic tasks, PVT, and processing speed in language and logic tasks. The decrease in improvement compared to a high dose shows that cerebral cellular creatine uptake and the improvement effect during sleep deprivation are dose-dependent. As the administered dose of 0.2 g/kg of creatine is safe, future studies could focus on adding additional components or making modifications to increase cellular uptake and enhance the effect. Furthermore, the findings of our study provide a basis for further research to determine the specific dosage for different population groups.

## Figures and Tables

**Table 1:** Outcome of cognitive tasks and scales. Mean number of correct results, processing time and intra-individual percentual changes versus baseline (6 pm) after creatine or placebo. Changes in Karolinska Sleepiness Scale (KSS) and fatigue scale before (bef.) and after (aft.) each session are shown below. Data are given ± SD.

**Task**		**No. of Trials**		**Number of Correct Results (Score)**																					
				**6 pm**						**12 am**					**2 am**						**4 am**				
				**Placebo**			**Creatine**			**Placebo**		**Creatine**			**Placebo**			**Creatine**			**Placebo**		**Creatine**		
WMT		22		16.4 ± 4.4			16.6 ± 4.2			15.7 ± 4.3		15.7 ± 4.7			13.6 ± 4.4			13.7 ± 5.0			12.5 ± 5.6		13.2 ± 5.2		
vs. 6 pm (%)										−4.4%		−5.8%			−17.2% *			−17.6% *			−23.9% **		−20.5% *		
Digit span		12		8.0 ± 2.5			8.0 ± 2.8			7.6 ± 2.3		6.7 ± 2.4			6.4 ± 2.7			7.2 ± 2.8			5.8 ± 2.5		7.0 ± 2.7		
vs. 6 pm (%)										−6.7%		−15.6%			−19.8% ^+^			−11.8%			−27.2% ^+^		−13.0%		
N-back		42		78 ± 20%			75.9 ± 21%			75.0 ± 24.7%		76.3 ± 19%			75.5 ± 23.5%			77.5 ± 21%			74.4 ± 23.6%		77.5 ± 18%		
vs. 6 pm (%)										−4.2%		4.0%			−6.7%			5.6%			−4.9%		5.5%		
Language		21		16.4 ± 3.1			16.0 ± 2.7			16.3 ± 2.7		17.1 ± 2.2			16.4 ± 3.0			16.2 ± 2.3			16.3 ± 2.5		15.8 ± 2.9		
vs. 6 pm (%)										−0.4%		7.3% ^+^			−0.2%			1.7%			−0.8%		−1.1%		
Logic		17		13.9 ± 2.1			14.2 ± 1.6			13.2 ± 2.4		14.2 ± 1.8			13.1 ± 2.8			14.2 ± 2.8			12.6 ± 3.3		14.0 ± 2.2		
vs. 6 pm (%)										−5.0% ^+^		0.0%			−5.9% ^+^			0.0%			−9.7% ^+^		−1.5%		
Numeric		9		7.4 ± 1.5			7.1±1.6			7.4 ± 1.5		7.9 ± 1.4			7.8 ± 1.8			7.8 ± 1.7			7.0 ± 2.3		7.3 ± 1.7		
vs. 6 pm (%)										−0.5%		10.1% ^+^			5.1%			8.7% ^+^			−6.0%		1.9%		
**Task**		**No. of Trials**		**Processing Time (t)**																					
				**6 pm**						**12 am**					**2 am**						**4 am**				
				**Placebo**			**Creatine**			**Placebo**		**Creatine**			**Placebo**			**Creatine**			**Placebo**		**Creatine**		
WMT		22		110 ± 34 s			111 ± 37 s			121 ± 44 s		113 ± 45 s			112 ± 32 s			120 ± 42 s			105 ± 33 s		120 ± 66 s		
vs. 6 pm (%)										9.5% ^+^		2.1%			2.1%			8.3%			−4.6%		8.2%		
Digit SPAN		12		27 ± 12 s			24 ± 11 s			30 ± 12 s		25 ± 7 s			27 ± 11 s			27 ± 9.2 s			26 ± 13 s		25 ± 8 s		
vs. 6 pm (%)										11.1%		0.5%			−1.0%			9.3%			−5.2%		2.8%		
Language		21		222 ± 78 s			228 ± 68 s			229 ± 72 s		202 ± 66 s			218 ± 70 s			230 ± 104 s			215 ± 72 s		197 ± 60 s		
vs. 6 pm (%)										3.1%		−11.5% ^+^			−1.7%			0.8%			−3.3%		−13.5% ^+^		
Logic		17		265 ± 97 s			244 ± 55 s			261 ± 78 s		226 ± 68 s			253 ± 70 s			228 ± 60 s			230 ± 58 s		204 ± 43 s		
vs. 6 pm (%)										−1.4%		−7.3% ^+^			−4.7%			−6.6%			−13.3% ^+^		−16.3% *		
Numeric		9		229 ± 102 s			217 ± 117 s			196 ± 74 s		189 ± 91			209 ± 89 s			215 ± 81			226 ± 91 s		221 ± 97		
vs. 6 pm (%)										−14.8% ^+^		−12.8%			−8.9%			−0.7%			−1.5%		2.0%		
**Karolinska Sleepiness Scale (KSS)**																									
	**6 pm**							**12 am**						**2 am**						**4 am**					
	**Placebo**				**Creatine**			**Placebo**			**Creatine**			**Placebo**			**Creatine**			**Placebo**				**Creatine**	
	**bef.**		**aft.**		**bef.**	**aft.**		**bef.**	**aft.**		**bef.**		**aft.**	**bef.**		**aft.**	**bef.**		**aft.**	**bef.**		**aft.**		**bef.**	**aft.**
**Score**	2.5		2.7		2.5	2.7		4.2	4.5		4.0		4.3	5.9		6.1	5.3		6.0	7.0		7.0		6.5	6.8
aft. vs. bef.	0.2				0.2			0.2			0.3			0.3			0.7			0				0.3	
vs. 6 pm (%)								69% ^c^			58% ***			135% ^h^			117% ^e^			173% ^j^				155% ^g^	
**Inverted Samn & Perelli Fatigue score (FAT)**																									
	**6 pm**							**12 am**						**2 am**						**4 am**					
	**Placebo**				**Creatine**			**Placebo**			**Creatine**			**Placebo**			**Creatine**			**Placebo**				**Creatine**	
	**bef.**		**aft.**		**bef.**	**aft.**		**bef.**	**aft.**		**bef.**		**aft.**	**bef.**		**aft.**	**bef.**		**aft.**	**bef.**		**aft.**		**bef.**	**aft.**
**Score**	6.7		7.3		5.6	6.0		8.7	9.5		7.9		8.3	11.2		12.8	10.1		11.8	14.1		14.3		12.9	13.9
aft. vs. bef.	1.1				0.4			0.8			0.4			1.6			1.7			0.4				0.9	
vs. 6 pm (%)								39% **			45% **			82% ^c^			100% ^c^			115% ^e^				148% ^f^	

^+^ *p* = values of 0.0063 ≤ *p* ≤ 0.05, that did not survive Bonferroni correction, * *p* = values of *p* ≤ 0.0064, that survived Bonferroni correction, ** *p* = values of *p* ≤ 0.0005, *** *p* = values of *p* ≤ 0.00005, ^c^ *p* ≤ 5.0 × 10^−8^, ^e–h^ *p* ≤ 5.0 × 10^−10^ to 5.0 × 10^−13^ in decadal steps and ^j^ *p* ≤ 5.0 × 10^−15^.

**Table 2:** Results in Psychomotor Vigilance Tests (PVTs) of reaction time (RT) and reaction time distribution (RTD) in the placebo and creatine sessions. The tests lasted 8 min. Trials with reaction times ≥ 1000 ms were considered to be lapses.

**PVT (Placebo)**								
	**RTD**	**Mean RT**	**Median RT**	**Minimum RT**	**Maximum RT**	**Fastest 10%**	**Slowest 10%**	**Speed**
6 pm	38 ± 13 ms	280 ± 26 ms	269 ± 23 ms	200 ± 22 ms	479 ± 76 ms	216 ± 17 ms	391 ± 52 ms	3.8 ± 0.3 1/s
12 am	42 ± 10 ms	292 ± 33 ms	281 ± 31 ms	203 ± 27 ms	569 ± 231 ms	224 ± 23 ms	415 ± 64 ms	3.6 ± 0.4 1/s
vs. 6 pm	12 ± 4% ^+^	4 ± 1% *	4 ± 1% *	2 ± 2%	13 ± 8% ^+^	4 ± 1% *	5 ± 2% ^+^	−3 ± 1% *
2 am	44 ± 8 ms	303 ± 36 ms	289 ± 34 ms	210 ± 19 ms	597 ± 248 ms	228 ± 20 ms	454 ± 93 ms	3.5 ± 0 1/s
vs. 6 pm	15.8 ± 8% *	8.3 ± 2% ***	7.3 ± 2% **	5.0 ± 2% ^+^	19.8 ± 8% ^+^	5.1 ± 1% **	14.1 ± 3% **	−6.5 ± 1% **
4 am	45 ± 8	316 ± 37	299 ± 36	211 ± 17	709 ± 354	229 ± 20	509 ± 118	3.3 ± 01/s
vs. 6 pm	18.4 ± 5% **	13.1 ± 2% ^b^	10.8 ± 2% ^a^	6.1 ± 2% *	41.9 ± 13% *	6.1 ± 1% ^a^	26.8 ± 5% ***	−9.5 ± 1% ^b^
vs. 6 pm Pooled	15.5 ± 2.7% ^b^	8.4 ± 1.0% ^g^	7.4 ± 1.0% ^f^	4.5% ± 1.1 **	24.9% ± 6.0 **	4.9% ± 0.7% ^f^	15.1 ± 2.3% ^d^	−6.4 ± 0.8% ^g^
**PVT (Creatine)**								
	**RTD**	**Mean RT**	**Median RT**	**Minimum RT**	**Maximum RT**	**Fastest 10%**	**Slowest 10%**	**Speed**
6 pm	40 ± 10 ms	281 ± 22 ms	270 ± 21 ms	199 ± 22 ms	500 ± 126 ms	218 ± 17 ms	396 ± 49 ms	3.7 ± 0.3 1/s
12 am	42 ± 9 ms	292 ± 32 ms	282 ± 33 ms	205 ± 26 ms	584 ± 325 ms	224 ± 21 ms	437 ± 120 ms	3.6 ± 0.4 1/s
vs. 6 pm	4.3 ± 2%	4.2 ± 1% ^+^	3.9 ± 1% ^+^	4.8 ± 1%	6.2 ± 3%	2.6 ± 1%	6.1 ± 1% ^+^	−3.1 ± 1% ^+^
2 am	43 ± 8 ms	300 ± 70 ms	293 ± 41 ms	210 ± 21 ms	638 ± 383	229 ± 22 ms	497 ± 293 ms	3.4 ± 0.4 1/s
vs. 6 pm	8.5 ± 4% ^+^	7.4 ± 1% **	6.8 ± 1% *	5.7 ± 3% ^+^	14.7 ± 4%	4.6 ± 2% *	12.5 ± 2%	−5.7 ± 1% *
4 am	43 ± 11 ms	306 ± 61 ms	296 ± 49 ms	214 ± 26 ms	673 ± 396 ms	231 ± 27 ms	481 ± 161 ms	3.4 ± 0.5 1/s
vs. 6 pm	8.1 ± 4%	9.4 ± 2% **	8.0 ± 2% *	6.6 ± 3% ^+^	22.9 ± 9% ^+^	5.3 ± 2% *	15.9 ± 4% *	−6.6 ±1% **
vs. 6 pm Pooled	7.0 ± 2% *	7.0 ± 1% ^c^	6.2 ± 1% ^b^	5.7 ± 4% **	14.6 ± 4% *	4.2 ± 1% ***	11.5 ± 2% ^c^	−5.1 ± 1% ^b^
**PVT (Creatine vs. Placebo)**								
**vs. 6 pm**	**RTD**	**Mean RT**	**Median RT**	**Minimum RT**	**Maximum RT**	**Fastest 10%**	**Slowest 10%**	**Speed**
12 am	−7.6 ± 5%	0.2 ± 1.7%	−0.6 ± 1.9%	1.5 ± 2.5%	−10.3 ± 10.7%	−1.2 ± 1.7%	1.0 ± 3.3%	−0.1 ± 1.5%
2 am	−9.8 ± 6.3%	−1.1 ± 2.2%	−0.6 ± 2.1%	−0.2 ± 2.8%	−9.0 ± 10.0%	−0.7 ± 1.9%	−2.0 ± 4.6%	0.6 ± 1.8%
4 am	−10.1 ± 6.7%	−3.1 ± 2.8%	−2.5 ± 2.6%	−0.8 ± 2.5%	−21.5 ± 16.1%	−0.5 ± 1.7%	−10.1 ± 5.9%	2.2 ± 2.1%
Pooled	−9.2 ± 3.4% ^+^	−1.3 ± 1.2%	−1.3 ± 1.3%	0.2 ± 1.5%	−13.6 ± 7.3%	−0.8 ± 1.0%	−3.7 ± 2.8%	0.9 ± 1.1%

^+^ *p* = values of 0.0063 ≤ *p* ≤ 0.05, that did not survive Bonferroni correction, * *p* = values of *p* ≤ 0.0064, that survived Bonferroni correction, ** *p* = values of *p* ≤ 0.0005, *** *p* = values of *p* ≤ 0.00005, ^a–d^ *p* ≤ 5.0 × 10^−6^ to 5.0 × 10^−9^ and ^f,g^ *p* ≤ 5.0 × 10^−11^ to 5.0 × 10^−12^ in decadal steps.

**Table 3:** Outcome of cognitive tasks and scales. Mean number of correct results, processing time and intra-individual percentual changes of creatine versus placebo. Changes in Karolinska Sleepiness Scale (KSS) and fatigue scale are shown below. Data are given as mean ± SD.

**Creatine Versus Placebo**							
**Task**	**No. of Trials**	**Number of Correct Results (Score) vs. 6 pm (%)**					
		**12 am**		**2 am**		**4 am**	**All 3 Time Points Pooled**
WMT	22	−5.0% ± 5.4%		−4.4% ± 6.4%		0.7% ± 0.3%	−2.9% ± 3.4%
Digit span	12	−19.5% ± 12.0%		15.0% ± 13.0%		8.4% ± 11.1%	1.2% ± 7.1%
N-back	42	3.8% ± 5.9%		6.4% ± 6.1%		5.5% ± 6.0%	5.2% ± 3.5%
Language	21	5.1% ± 4.8%		−0.5% ± 5.3%		−1.5% ± 0.3%	1.0% ± 2.8%
Logic	17	4.4% ± 3.2%		5.7% ± 3.2%		8.1% ± 4.4%	6.1% ± 2.1% *
Numeric	9	8.9% ± 5.8%		2.4% ± 4.6%		6.9% ± 4.6%	6.2% ± 2.9% ^+^
**Task**	**No. of trials**	**Processing Time (t) vs. 6 pm (%)**					
		**12 am**			**2 am**	**4 am**	**all 3 time points pooled**
WMT	22	−8.5% ± 4.5%			4.3% ± 5.1%	7.9% ± 10.3%	1.2% ± 4.2%
Digit SPAN	12	−17.4% ± 13.7%			3.0% ± 10.4%	2.8% ± 16.8%	−3.9% ± 8.1%
Language	21	−20.1% ± 9.0% ^+^			−0.8% ± 10.2%	−16.2% ± 10.4%	−12.3% ± 5.8% ^+^
Logic	17	−5.7% ± 4.9%			−2.6% ± 7.1%	−2.7% ± 6.6%	−3.6% ± 3.6%
Numeric	9	0.2% ± 6.8%			1.2% ± 8.9%	0.7% ± 7.2%	4.4% ± 0.7%
**Karolinska Sleepiness Scale (KSS)**							
**Score**			**12 am**		**2 am**	**4 am**	**All 3 Time Points Pooled**
**vs. 6 pm (%)**			−5.8% ± 2.1%		−4.9% ± 2.5%	2.8% ± 1.5%	−2.6 ± 0.7%
**Inverted Samn & Perelli Fatigue score (FAT)**							
**Score**			**12 am**		**2 am**	**4 am**	**all 3 time points pooled**
**vs. 6 pm (%)**			−15.9 ± 16.0%		−6.6 ± 5.9%	6.0 ± 8.0%	−5.5 ± 3.4%

^+^ *p* = values of 0.0063 ≤ *p* ≤ 0.05, that did not survive Bonferroni correction, * *p* = values of *p* ≤ 0.0064, that survived Bonferroni correction.

**Table 4:** Pearson’s correlation coefficients (*r_p_*_,_ *p*-value) between changes in FAT, KSS score and cognitive score after creatine or placebo administration when pooled at all 3 time points (12 am, 2 am, 4 am).

**Placebo**							
	**PVT (RT)**	**WMT**	**SPAN**	**N-Back**	**Language**	**Logic**	**Numeric**
**FAT**	0.27 ^+^	−0.02	0.15	−0.10	−0.07	0.038	0.01
**KSS**	0.43 ***	−0.18	−0.05	−0.11	−0.05	0.049	0.013
**Creatine**							
	**PVT (RT)**	**WMT**	**SPAN**	**N-Back**	**Language**	**Logic**	**Numeric**
**FAT**	0.20	−0.17	0.01	−0.001	−0.07	−0.17	−0.27 ^+^
**KSS**	0.25 ^+^	−0.24	0.06	0.20	−0.14	−0.12	−0.18
**Creatine versus Placebo**							
	**PVT (RT)**	**WMT**	**SPAN**	**N-Back**	**Language**	**Logic**	**Numeric**
**FAT**	0.28 ^+^	0.01	0.04	−0.001	−0.14	−0.01	0.02
**KSS**	0.19	−0.08	0.08	0.02	−0.15	−0.06	0.06

^+^ *p* = values of 0.0063 ≤ *p* ≤ 0.05, that did not survive Bonferroni correction, *** *p* = values of *p* ≤ 0.00005.

## Data Availability

The original data presented in the study are openly available in Zenodo at https://zenodo.org/records/18942690 (accessed on 6 April 2026).

## Supplementary Materials

The supporting information can be downloaded at https://www.mdpi.com/article/10.3390/nu18081192/s1. Figure S1: Baseline (6 pm) related changes in cognitive performance during sleep deprivation under creatine, placebo and creatine versus placebo at 0 pm, 2 am and 4 am. Shown are changes in language, logic, numeric, forward Digit Span (SPAN), Word Memory Tasks (WMT) and Psychomotor Vigilance Tests (PVT, Reaction Time RT). Significance levels are presented by ^+^ *p*_27_ ≤ 0.05, * *p*_27_ ≤ 0.005, ** *p*_27_ ≤ 0.0005, *** *p*_27_ ≤ 0.00005 and ^c^ *p*_27_ ≤ 5.0 × 10^−7^. Bars represent standard errors (SE). Figure S2: Baseline (6 pm) related changes in cognitive performance for female and male subjects during sleep deprivation under creatine or placebo at 0 pm, 2 am and 4 am. Shown are changes in language, logic, numeric, forward Digit Span (SPAN), Word Memory Tasks (WMT) and Psychomotor Vigilance Tests (PVT, Reaction Time RT). Significance levels are presented by ^+^ *p*_27_ ≤ 0.05, * *p*_27_ ≤ 0.005, ** *p*_27_ ≤ 0.0005. Bars represent standard errors (SE). Figure S3: Baseline (6 pm) related changes in cognitive performance for vegetarian and non-vegetarian subjects during sleep deprivation under creatine or placebo at 0 pm, 2 am and 4 am. Shown are changes in language, logic, numeric, forward Digit Span (SPAN), Word Memory Tasks (WMT) and Psychomotor Vigilance Tests (PVT, Reaction Time RT). Significance levels are presented by ^+^ *p*_27_ ≤ 0.05, * *p*_27_ ≤ 0.005, ** *p*_27_ ≤ 0.0005. Bars represent standard errors (SE). Figure S4: Baseline (6 pm) related changes in cognitive performance for females, males, vegetarians und non-vegetarians during sleep deprivation under creatine versus placebo at 0 pm, 2 am and 4 am. Shown are changes in language, logic, numeric, forward Digit Span (SPAN), Word Memory Tasks (WMT) and Psychomotor Vigilance Tests (PVT, Reaction Time RT). Significance levels are presented by ^+^ *p*_27_ ≤ 0.05. Bars represent standard errors (SE). Table S1: Outcome of cognitive tasks and scales for female participants. Mean number of correct results, processing time and intra-individual percentual changes versus baseline (6 pm) after administration of creatine or placebo. Changes in Karolinska Sleepiness Scale (KSS) and Fatigue scale before (bef.) and after (aft.) each session are shown below. Data are given ± SD. Table S2: Outcome of cognitive tasks and scales for male participants. Mean number of correct results, processing time and intra-individual percentual changes versus baseline (6 pm) after administration of creatine or placebo. Changes in Karolinska Sleepiness Scale (KSS) and Fatigue scale before (bef.) and after (aft.) each session are shown below. Data are given ± SD. Table S3: Outcome of cognitive tasks and scales for vegetarian participants. Mean number of correct results, processing time and intra-individual percentual changes versus baseline (6 pm) after administration of creatine or placebo. Changes in Karolinska Sleepiness Scale (KSS) and Fatigue scale before (bef.) and after (aft.) each session are shown below. Data are given ± SD. Table S4: Outcome of cognitive tasks and scales for non-vegetarian participants. Mean number of correct results, processing time and intra-individual percentual changes versus baseline (6 pm) after administration of creatine or placebo. Changes in Karolinska Sleepiness Scale (KSS) and Fatigue scale before (bef.) and after (aft.) each session are shown below. Data are given ± SD. Table S5: Outcome of cognitive tasks and scales for female participants. Mean number of correct results, processing time and intra-individual percentual changes of creatine versus placebo. Changes in Karolinska Sleepiness Scale (KSS) and Fatigue scale are shown below. Table S6: Outcome of cognitive tasks and scales for male participants. Mean number of correct results, processing time and intra-individual percentual changes of creatine versus placebo. Changes in Karolinska Sleepiness Scale (KSS) and Fatigue scale are shown below. Table S7: Outcome of cognitive tasks and scales for vegetarian participants. Mean number of correct results, processing time and intra-individual percentual changes of creatine versus placebo. Changes in Karolinska Sleepiness Scale (KSS) and Fatigue scale are shown below. Table S8: Outcome of cognitive tasks and scales for non-vegetarian participants. Mean number of correct results, processing time and intra-individual percentual changes of creatine versus placebo. Changes in Karolinska Sleepiness Scale (KSS) and Fatigue scale are shown below. Table S9: Results in psychomotor vigilance tests (PVT) of reaction time (RT) and reaction time distribution (RTD) in the placebo and creatine session for female participants. The tests lasted 8 min. Trials with reaction times ≥ 1000 ms were considered as lapses. Table S10: Results in psychomotor vigilance tests (PVT) of reaction time (RT) and reaction time distribution (RTD) in the placebo and creatine session for male participants. The tests lasted 8 min. Trials with reaction times ≥ 1000 ms were considered as lapses. Table S11: Results in psychomotor vigilance tests (PVT) of reaction time (RT) and reaction time distribution (RTD) in the placebo and creatine session for vegetarian participants. The tests lasted 8 min. Trials with reaction times ≥ 1000 ms were considered as lapses. Table S12: Results in psychomotor vigilance tests (PVT) of reaction time (RT) and reaction time distribution (RTD) in the placebo and creatine session for non-vegetarian participants. The tests lasted 8 min. Trials with reaction times ≥ 1000 ms were considered as lapses. Table S13: Pearson’s correlation coefficients (*r_p_*, *p*-value) between changes in FAT, KSS score and cognitive score in females and males after creatine or placebo administration when pooled at all 3 timepoints (0 pm, 2 am, 4 am). Table S14: Pearson’s correlation coefficients (*r_p_*, *p*-value) between changes in FAT und KSS score and cognitive score in vegetarians and non-vegetarians after creatine or placebo administration when pooled at all 3 timepoints (0 pm, 2 am, 4 am).
