# Supplementary material for: Single-Dose Creatine Reduces Sleep Deprivation-Induced Deterioration in Cognitive Performance
Source: Nutrients. 2026 Apr 10;18(8):1192. doi: 10.3390/nu18081192 (PMC13119191; doi:10.3390/nu18081192)
Supplement: Supplementary file 1 [file nutrients-18-01192-s001.zip › nutrients-4222199-supplementary.pdf]

Supplement

To quantify the net effect of each condition, intra-individual changes relative to baseline (6 pm) were calculated as normalized differences:

$$\overline{\Delta_t} = \frac{1}{N}\frac{\sum_{i=0}^{N} X_{i,t} - X_{i,baseline}}{\sum_{i=0}^{N} X_{i,baseline}} \quad , \quad t \in \{0\text{pm}, 2\text{am}, 4\text{am}\} \qquad \text{[S1]}$$

were pooled across three post-supplementation intervals (0pm, 2am, and 4am) by using a two-tailed paired-sample tests where $X_{i,t}$ represents the score value of subject *i* at time point ***t*** and $X_{i,baseline}$ denotes the baseline value measured at 6pm.

To calculate the net protective effect of creatine against sleep-deprivation-induced cognitive decline, a difference-in-differences (DiD) analysis was performed by subtracting baseline-corrected change in placebo $\Delta Pl_{i,t}$ from the corresponding change under creatine $\Delta Cr_{i,t}$:

$$\overline{\Delta_t^{DiD}} = \frac{1}{N}\sum_{i=1}^{N}(\Delta_{i,t}^{Cr} - \Delta_{i,t}^{Pl}) \qquad \text{[S2]}$$

Figure S1

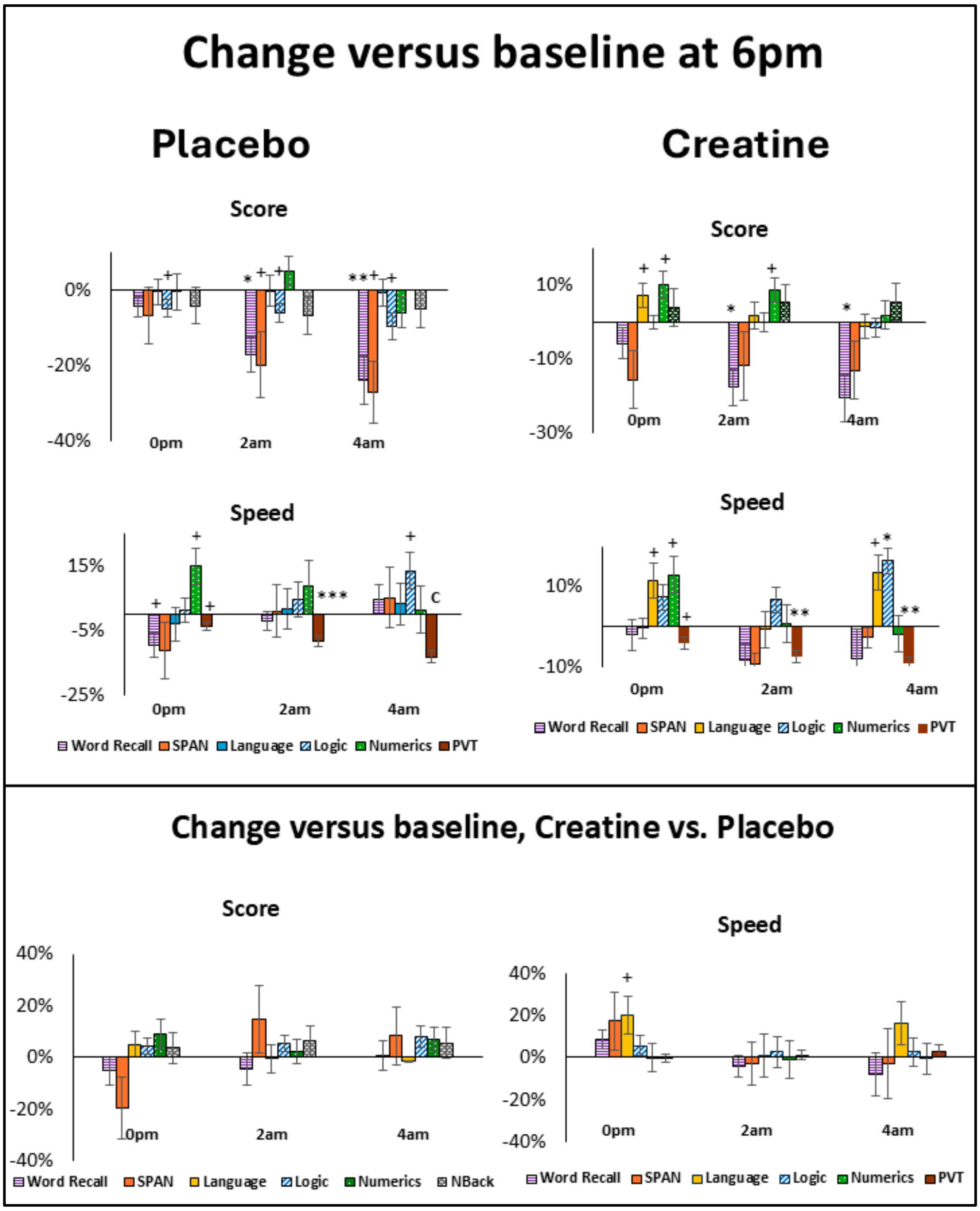

Figure S1 Baseline (6pm) related changes in cognitive performance during sleep deprivation under creatine, placebo and creatine versus placebo at 0pm, 2am and 4am. Shown are changes in language, logic, numeric, forward Digit Span (SPAN), Word Memory Tasks (WMT) and Psychomotor Vigilance Tests (PVT, Reaction Time TR). Significance levels are presented by $^{+}$ $p_{27} \leq 0.05$, * $p_{27} \leq 0.005$, ** $p_{27} \leq 0.0005$, *** $p_{27} \leq 0.00005$ and ^{c} $p_{27} \leq 5.0 \times 10^{-7}$. Bars represent standard errors (SE)

Figure S2

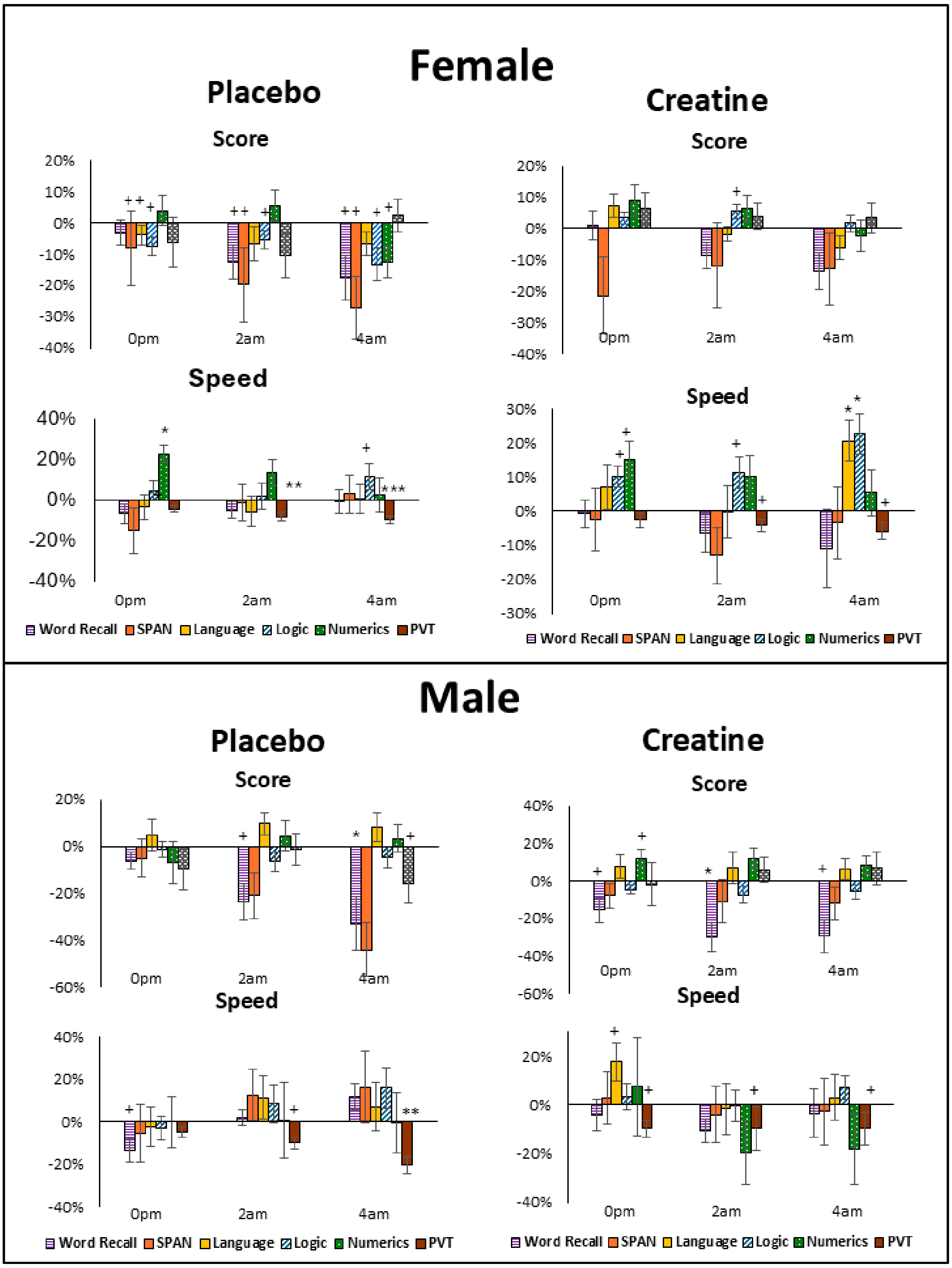

Figure S2 Baseline (6pm) related changes in cognitive performance for female and male subjects during sleep deprivation under creatine or placebo at 0pm, 2am and 4am. Shown are changes in language, logic, numeric, forward Digit Span (SPAN), Word Memory Tasks (WMT) and Psychomotor Vigilance Tests (PVT, Reaction Time TR). Significance levels are presented by $^{+}$ $p_{27} \leq 0.05$, * $p_{27} \leq 0.005$, ** $p_{27} \leq 0.0005$. Bars represent standard errors (SE)

Figure S3

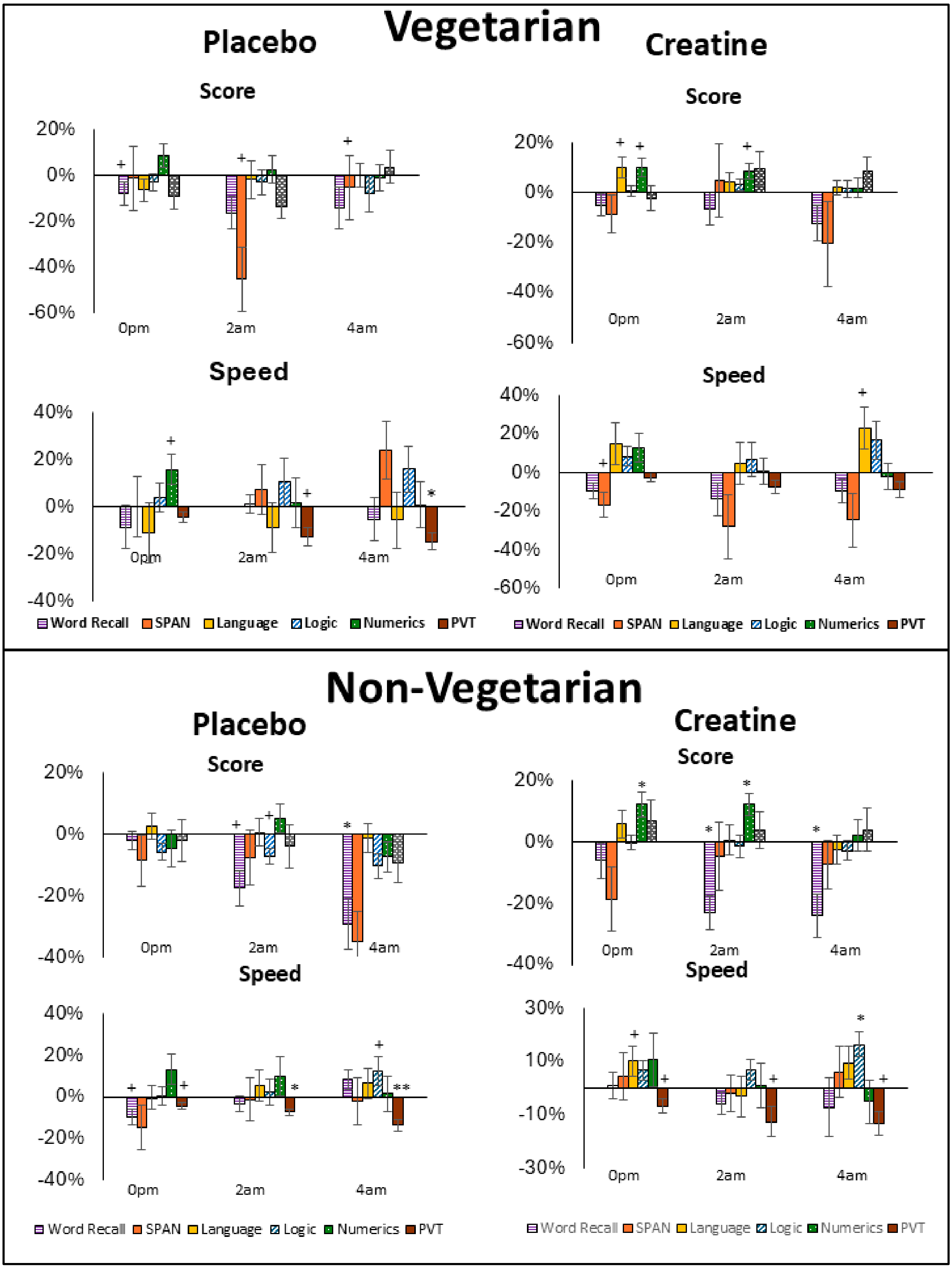

Figure S3 Baseline (6pm) related changes in cognitive performance for vegetarian and non-vegetarian subjects during sleep deprivation under creatine or placebo at 0pm, 2am and 4am. Shown are changes in language, logic, numeric, forward Digit Span (SPAN), Word Memory Tasks (WMT) and Psychomotor Vigilance Tests (PVT, Reaction Time TR). Significance levels are presented by $^{+}$ $p_{27} \leq 0.05$, * $p_{27} \leq 0.005$, ** $p_{27} \leq 0.0005$. Bars represent standard errors (SE)

Figure S4

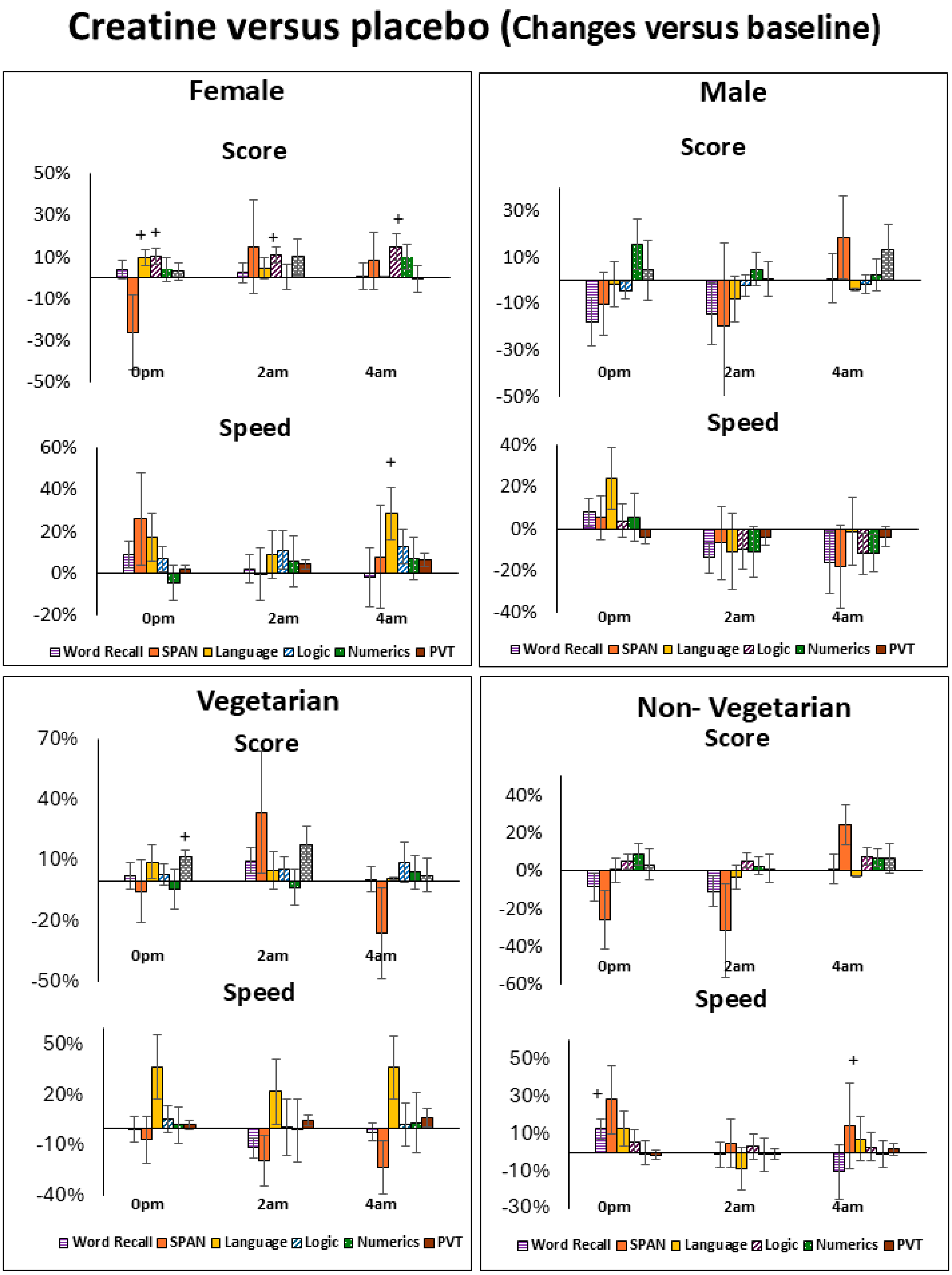

Figure S4 Baseline (6pm) related changes in cognitive performance for females, males, vegetarians und non-vegetarians during sleep deprivation under creatine versus placebo at 0pm, 2am and 4am. Shown are changes in language, logic, numeric, forward Digit Span (SPAN), Word Memory Tasks (WMT) and Psychomotor Vigilance Tests (PVT, Reaction Time TR). Significance levels are presented by $^{+}$ $p_{27} \leq 0.05$. Bars represent standard errors (SE)

Table S1

Outcome of cognitive tasks and scales for female participants. Mean number of correct results, processing time and intra-individual percentual changes versus baseline (6 pm) after administration of creatine or placebo. Changes in Karolinska Sleepiness Scale (KSS) and Fatigue scale before (bef.) and after (aft.) each session are shown below. Data are given ±SD.

Task	No. of trials	Number of correct results (Score)							
		6 pm		0 am		2 am		4 am	
		Placebo	Creatine	Placebo	Creatine	Placebo	Creatine	Placebo	Creatine
WMT	22	16.4±3.8	16.5±4.0	15.9 ±4.1	16.7±4.1	14.4 ±4.6	15.1±4.2	13.5 ±5.5	14.2±5.5
vs. 6pm (%)				-3.2%	1.0%	-12.5%$^{+}$	-8.4%	-17.6%$^{+}$	-13.4%
Digit span	12	7.8±2.5	7.6±3.1	7.3 ±2.3	6.0±2.5	6.0 ±2.9	6.9±2.6	6.2 ±1.7	6.7±3.1
vs. 6pm (%)				-8.0%	-21.5%	-22.7%$^{+}$	-10.7%	-19.7%$^{+}$	-12.3%
N-back	42	79±19%	76±17%	79.8±19.0%	80.8±13%	75.9±20.9%	79.0±18%	81.6±13.2%	78.5±15%
vs. 6pm (%)				-6.3%	6.3%	-10.5%	4.0%	2.3%	3.3%
Language	21	17.0±2.4	16.4±2.1	16.4 ±2.7	17.6±1.9	15.9 ±3.3	16.1±2.3	15.9 ±3.0	15.4±3.3
vs. 6pm (%)				-3.8%	7.2%	-6.5%	-1.8%	-6.6%$^{+}$	-6.1%
Logic	17	13.8±1.9	13.9±1.5	12.8 ±2.1	14.4±1.5	13.1 ±2.1	14.6±2.0	12.0 ±3.1	14.1±1.7
vs. 6pm (%)				-7.7%$^{+}$	3.4%	-5.5%$^{+}$	5.5%$^{+}$	-13.2%$^{+}$	1.7%
Numeric	9	7.5±1.4	7.4±1.1	7.8 ±1.1	8.0±1.0	7.9 ±1.4	7.8±1.1	6.5 ±2.3	7.2±1.6
vs. 6pm (%)				3.9%	8.8%	5.5%	6.4%	-12.6%$^{+}$	-2.4%

Task	No. of trials	Processing Time (t)							
		6 pm		0 am		2 am		4 am	
		placebo	creatine	placebo	creatine	placebo	creatine	placebo	creatine
WMT	22	110 ±37 s	109±34 s	118 ±42 s	109±29 s	116 ±37 s	116±34 s	111 ±35 s	121±60 s
vs. 6 pm (%)				6.8%	0.7%	5.0%	6.2%	0.7%	11.0%
Digit SPAN	12	27 ±10 s	25±12 s	31 ±15 s	26±8	28 ±11 s	28±11	37 ±11 s	26±8
vs. 6 pm (%)				15.1%	2.7%	1.3%	13.0%	-3.0%	3.3%
Language	21	217 ±70 s	229±69 s	225 ±77 s	212±74 s	229 ±84 s	229±102 s	215 ±86 s	181±48 s
vs. 6 pm (%)				3.6%	-7.1%	5.6%	0.2%	-0.6%	-20.7%*
Logic	17	255 ±93 s	246±57 s	243 ±76 s	222±62 s	251 ±72 s	218±54 s	226 ±52 s	190±32 s
vs. 6 pm (%)				-4.7%	-10.1%$^{+}$	-1.7%	-11.3%$^{+}$	-11.3%$^{+}$	-22.7%*
Numeric	9	256 ±99 s	254±125 s	197 ±78 s	215±100 s	221 ±98 s	228±98 s	249 ±100 s	240±115 s
vs. 6 pm (%)				-22.7%*	-15.2%$^{+}$	-13.4%*	-10.0%	-2.5%	-5.4%

Karolinska Sleepiness Scale (KSS)

	6 pm				0 am				2 am				4 am			
	Placebo		Creatine		Placebo		Creatine		Placebo		Creatine		Placebo		Creatine	
	bef.	aft.	bef.	aft	bef.	aft.	bef.	aft.	bef.	aft.	bef.	aft.	bef.	aft.	bef.	aft.
**Score**	2.4	2.7	2.6	2.7	3.9	4.2	3.8	4.1	5.9	6.2	5.2	5.9	6.8	6.8	6.4	6.8
aft vs. bef	0.3		0.8		0.3		0.4		0.4		2.2		0		0.6	
vs. 6 pm (%)					61%**		50%*		140% ^{c}		108% ^{a}		169% ^{c}		146% ^{b}	

Inverted Samn & Perelli Fatigue score (FAT)

	6 pm				11 pm				2 am				4 am			
	Placebo		Creatine		Placebo		Creatine		Placebo		Creatine		Placebo		Creatine	
	bef	aft.	bef.	aft.	bef.	aft.	bef.	aft	bef.	aft.	bef.	aft	bef.	aft.	bef.	aft.
**Score**	6.3	7.6	5.6	6.4	8.2	9.2	7.6	7.9	11.4	12.8	9.7	11.9	14.4	15.2	12.9	13.5
aft vs. bef	1.4		0.1		1.0		0.4		1.5		0.7		0.8		0.4	
vs. 6 pm (%)					33% $^{+}$		34%$^{+}$		83%**		92%**		122% ^{a}		139%^{a}	

$^{+}$p= values of 0.0063, ≤ p ≤ 0.05, that did not survive Bonferroni correction, * p= values of p ≤ 0.0064, that survived Bonferroni correction, ** p= values of p ≤ 0.0005, *** p= values of p ≤ 0.00005, $^{\mathbf{a–d}}$ p ≤ 5.0×10^{-6} to 5.0×10^{-9} in decadal steps.

Table S2
Outcome of cognitive tasks and scales for male participants. Mean number of correct results, processing time and intra-individual percentual changes versus baseline (6 pm) after administration of creatine or placebo. Changes in Karolinska Sleepiness Scale (KSS) and Fatigue scale before (bef.) and after (aft.) each session are shown below. Data are given ±SD.

Task	No. of trials	Number of correct results (Score)							
		6 pm		0 am		2 am		4 am	
		Placebo	Creatine	Placebo	Creatine	Placebo	Creatine	Placebo	Creatine
WMT	22	16.5±5.1	16.8±4.3	15.5±4.7	14.2±5.1	12.6±3.8	11.8±5.3	11.1±5.4	11.8±4.5
vs. 6pm (%)				-6.1%	-15.4%$^{+}$	-23.7%$^{+}$	-29.9%*	-32.8%*	-29.4%$^{+}$
Digit span	12	8.3±2.6	8.4±2.2	7.9±2.1	7.8±2.0	7.0±2.3	7.6±3.1	5.3±3.2	7.5±2.0
vs. 6pm (%)				-5.0%	-7.9%	-21.0%	-10.8%	-44.0%	-12.1%
N-back	42	76±23%	71±29%	68.5±25.7%	70±25%	75.3±27.6%	75±25%	64.3±31.3%	76±23%
vs. 6pm (%)				-9.8%	-1.8%	-1.1%	5.9%	-15.7%$^{+}$	6.8%
Language	21	15.6±3.7	15.3±3.3	16.3±2.8	16.5±2.4	17.1±2.4	16.4±2.4	16.8±1.5	16.3±2.2
vs. 6pm (%)				4.8%	7.6%	9.6%$^{+}$	7.1%	8.0%	6.5%
Logic	17	14.1±2.3	14.7±1.7	13.9±2.7	14.0±2.2	13.2±3.6	13.6±3.6	13.4±3.4	13.8±2.8
vs. 6pm (%)				-1.2%	-4.5%	-6.5%	-7.4%	-4.7%	-5.7%
Numeric	9	7.4±1.7	6.8±1.9	6.9±1.9	7.7±1.8	7.8±2.2	7.7±2.2	7.7±2.1	7.4±1.8
vs. 6pm (%)				-6.7%	12.2%$^{+}$	4.5%	12.2%	3.4%	8.5%

Task	No. of trials	Processing Time (t)							
		6 pm		0 am		2 am		4 am	
		placebo	creatine	placebo	creatine	placebo	creatine	placebo	creatine
WMT	22	109±31 s	114±41 s	124±48 s	119±61 s	107±22 s	127±50 s	96±27 s	118±74 s
vs. 6 pm (%)				13.4%$^{+}$	4.1%	-2.0%	10.8%	-12.1%	3.4%
Digit SPAN	12	27 ±15 s	24±8 s	28±9 s	23±5 s	25±11 s	25±4 s	24±15 s	24±6 s
vs. 6 pm (%)				5.5%	-2.8%	-12.6%	3.9%	-16.3%	2.7%
Language	21	230 ±87 s	226±65 s	235 ±64 s	186±47 s	203 ±38 s	230±106 s	213 ±48	219±67 s
vs. 6 pm (%)				2.3%	-17.7%$^{+}$	-11.5%	1.6%	-7.1%	-3.1%
Logic	17	279±100 s	240±51 s	287±73 s	232±75 s	255±67 s	240±66 s	235±66 s	223±49 s
vs. 6 pm (%)				2.8%	-3.2%	-8.5%	0.2%	-15.9%	-7.1%
Numeric	9	193±95 s	165±80 s	193±66 s	152±61 s	192±71 s	197±42 s	194±62 s	195±55 s
vs. 6 pm (%)				0.01%	-7.6%	-0.6%	19.7%	0.5%	18.1%

Karolinska Sleepiness Scale (KSS)																
	6 pm				0 am				2 am				4 am			
	Placebo		Creatine		Placebo		Creatine		Placebo		Creatine		Placebo		Creatine	
	bef.	aft.	bef.	aft	bef.	aft.	bef.	aft.	bef.	aft.	bef.	aft.	bef.	aft.	bef.	aft.
**Score**	2.7	2.6	2.3	2.6	4.6	4.8	4.2	4.5	5.8	6	5.4	6.0	7.3	7.1	6.5	6.9
aft vs. bef	0		0.3		0.3		0.3		0.3		0.7		-0.2		0.3	
vs. 6 pm (%)					77%*		64%$^{+}$		120%***		130%**		169% ^{b}		170%***	

Inverted Samn & Perelli Fatigue score (FAT)																
	6 pm				11 pm				2 am				4 am			
	Placebo		Creatine		Placebo		Creatine		Placebo		Creatine		Placebo		Creatine	
	bef	aft.	bef.	aft.	bef.	aft.	bef.	aft	bef.	aft.	bef.	aft	bef.	aft.	bef.	aft.
**Score**	6.3	6.9	5.4	5.3	9.5	10	8.3	8.8	11	12.8	10.6	11.7	13.7	13.4	13.0	14.3
aft vs. bef	0.7		-0.1		0.5		0.4		1.8		1.1		-0.3		1.3	
vs. 6 pm (%)					44%***		57%$^{+}$		81%**		111%**		105%***		161% ^{a}	

$^{+}$p= values of 0.0063, ≤ p ≤ 0.05, that did not survive Bonferroni correction, * p= values of $p \leq 0.0064$, that survived Bonferroni correction, ** p= values of $p \leq 0.0005$, *** p= values of $p \leq 0.00005$, $^{\mathbf{a}}$ $p \leq 5.0 \times 10^{-6}$, $^{\mathbf{b}}$ $p \leq 5.0 \times 10^{-7}$.

Table S3

Outcome of cognitive tasks and scales for vegetarian participants. Mean number of correct results, processing time and intra-individual percentual changes versus baseline (6 pm) after administration of creatine or placebo. Changes in Karolinska Sleepiness Scale (KSS) and Fatigue scale before (bef.) and after (aft.) each session are shown below. Data are given ±SD.

Task	No. of trials	Number of correct results (Score)							
		6 pm		0 am		2 am		4 am	
		Placebo	Creatine	Placebo	Creatine	Placebo	Creatine	Placebo	Creatine
WMT	22	18.9±1.7	18.6±2.2	17.3±2.4	17.6±2.2	15.8±4.0	17.2±2.7	16.2±3.5	16.0±3.2
vs. 6pm (%)				-8.2%	-4.9%	-16.5%+	-6.5%	-14.1%	-12.4%
Digit span	12	8.1±2.7	7.8±2.1	7.8±2.4	7.1±2.8	4.2±2.7	8.1±2.6	7.4±1.8	6.1±2.8
vs. 6pm (%)				-1.4%	-8.6%	-45.3%+	4.7%	-5.5%+	-20.5%
N-back	42	85.9±16%	79±15%	78.8± 26%	77±13%	75.5±19%	86±17%	86.5±11%	85±16%
vs. 6pm (%)				-9.4%	-2.3%	-13.7%	9.5%	3.6%	8.8%
Language	21	17.3±2.4	16.2±1.4	16.2±1.7	17.9±2.4	17.0±3.1	16.9±1.5	17.3±1.2	16.6±1.8
vs. 6pm (%)				-6.4%	10.3%+	-1.9%	4.1%	0%	2.1%
Logic	17	13.9±1.4	14.1±1.2	13.4±1.5	14.2±0.9	13.4±2.2	14.6±1.3	12.8±2.9	14.3±1.2
vs. 6pm (%)				-3.2%	0.8%	-3.2%	3.1%	-8.0%	1.6%
Numeric	9	7.4±1.3	7.1±1.5	8.1±1.1	7.9±1.4	7.6±1.4	7.8±1.7	7.3±1.8	7.3±1.7
vs. 6pm (%)				8.6%	10.1%+	2.5%	8.7%+	-1.2%	1.9%

Task	No. of trials	Processing Time (t)							
		6 pm		0 am		2 am		4 am	
		placebo	creatine	placebo	creatine	placebo	creatine	placebo	creatine
WMT	22	99±17 s	97±19 s	108±33 s	107±19 s	98±17 s	111±33 s	104±30 s	107±20
vs. 6 pm (%)				8.7%	9.4%	-1.2%	13.7%	5.3%	9.5%
Digit SPAN	12	24±13 s	18±5 s	24±7 s	22±5 s	22±11 s	25±11 s	18±6 s	25±5 s
vs. 6 pm (%)				0.0%	16.7%	-7.2%	27.9%	-23.9%	24.6%
Language	21	184±58 s	216±54 s	205±57 s	184±52 s	200±37 s	205±40 s	195±38 s	166±35 s
vs. 6 pm (%)				11.1%	-14.9%	8.7%	-4.9%	5.7%	-23.1%+
Logic	17	254±99 s	243±51 s	244±69 s	223±61 s	227±61 s	227±50 s	213±44 s	203±41 s
vs. 6 pm (%)				-3.8%	-8.4%	-10.3%	-6.6%	-15.8%	-16.6%
Numeric	9	208±102 s	217±117 s	172±78 s	189±91 s	205±107 s	215±81 s	206±81 s	221±97 s
vs. 6 pm (%)				-15.8%+	-12.8%	-1.6%	-0.7%	-0.8%	2.0%

Karolinska Sleepiness Scale (KSS)

	6 pm				0 am				2 am				4 am			
	Placebo		Creatine		Placebo		Creatine		Placebo		Creatine		Placebo		Creatine	
	bef.	aft.	bef.	aft	bef.	aft.	bef.	aft.	bef.	aft.	bef.	aft.	bef.	aft.	bef.	aft.
**Score**	2.6	3.2	2.3	2.3	4.6	5.0	3.8	4.3	7.0	7.3	5.5	6.2	7.6	7.3	7.0	7.3
aft vs. bef	0.7		0.0		0.4		0.4		0.3		0.7		-0.2		0.3	
vs. 6 pm (%)					65%**		7%		148% a		53%		157%***		87%+	

Inverted Samn & Perelli Fatigue score (FAT)

	6 pm				11 pm				2 am				4 am			
	Placebo		Creatine		Placebo		Creatine		Placebo		Creatine		Placebo		Creatine	
	bef	aft.	bef.	aft.	bef.	aft.	bef.	aft	bef.	aft.	bef.	aft	bef.	aft.	bef.	aft.
**Score**	5.6	6.4	5.3	5.0	8.2	9.2	7.2	7.8	12.4	13.9	10.2	12.1	15.6	15.9	14.0	14.4
aft vs. bef	0.9		-0.3		1.0		0.6		1.4		1.9		0.3		0.4	
vs. 6 pm (%)					45%+		50%		119%**		115%**		162%**		174%**	

+p= values of $0.0063 \le p \le 0.05$, that did not survive Bonferroni correction, * p= values of $p \le 0.0064$, that survived Bonferroni correction, ** p= values of $p \le 0.0005$, *** p= values of $p \le 0.00005$, [a] $p \le 5.0 \times 10^{-6}$.

Table S4

Outcome of cognitive tasks and scales for non-vegetarian participants. Mean number of correct results, processing time and intra-individual percentual changes versus baseline (6 pm) after administration of creatine or placebo. Changes in Karolinska Sleepiness Scale (KSS) and Fatigue scale before (bef.) and after (aft.) each session are shown below. Data are given ±SD.

Task	No. of trials	Number of correct results (Score)							
		6 pm		0 am		2 am		4 am	
		Placebo	Creatine	Placebo	Creatine	Placebo	Creatine	Placebo	Creatine
WMT	22	15.4±4.8	15.8±4.5	15.0±4.9	14.8±5.2	12.7±4.2	12.1±5.0	10.9±5.6	12.0±5.5
vs. 6pm (%)				-2.3%	-6.0%	-17.6%*	-23.2%*	-29.3%*	-24.1%*
Digit span	12	8.1±2.5	8.1±3.1	7.5±2.2	6.6±2.3	7.4±2.1	6.8±2.9	5.1±2.4	7.5±2.6
vs. 6pm (%)				-8.7%	-18.6%	-7.7%	-16.1%	-34.9%	-7.5%
N-back	42	74.9±21%	74.4±24	73.4±24%	76±22	75.7±25%	73±22	68.0±25%	74±19
vs. 6pm (%)				-2.0%	7.0%	-3.9%	3.8%	-9.1%	4.0%
Language	21	16.0±3.2	15.9±3.2	16.4±3.1	16.8±2.0	16.1±2.9	16.0±2.6	15.8±2.8	15.5±3.2
vs. 6pm (%)				2.5%	6.0%	0.6%	0.6%	-1.3%	-2.5%
Logic	17	14.0±2.3	14.3±1.8	13.2±2.7	14.2±2.1	13.0±3.1	14.1±3.3	12.5±3.4	13.9±2.6
vs. 6pm (%)				-5.7%	-0.4%	-7.2%$^+$	-1.4%	-10.4%	-2.8%
Numeric	9	7.5±1.3	7.0±1.5	7.2±1.1	7.8±1.6	7.9±1.4	7.8±1.8	7.0±1.8	7.1±1.9
vs. 6pm (%)				-4.7%	12.2%$^+$	5.3%	12.2%$^+$	-7.3%	2.2%

Task	No. of trials	Processing Time (t)							
		6 pm		0 am		2 am		4 am	
		placebo	creatine	placebo	creatine	placebo	creatine	placebo	creatine
WMT	22	115±39 s	117±41 s	126±47 s	116±53 s	119.4±35 s	124±45 s	105±34 s	126±78 s
vs. 6 pm (%)				9.8%$^+$	-0.9%	3.4%	5.8%	-8.4%	7.3%
Digit SPAN	12	28±12 s	27±11 s	32±14 s	26±7 s	29±11 s	28±7 s	28±14 s	26±8 s
vs. 6 pm (%)				14.8%	-4.5%	1.3%	2.3%	1.0%	-6.0%
Language	21	239±79 s	233±72 s	240±75 s	210±69 s	227±79 s	241±120 s	224±82 s	211±63 s
vs. 6 pm (%)				0.3%	-10.0%$^+$	-5.3%	3.2%	-6.5%	-9.5%
Logic	17	270±95 s	244±57 s	269±80 s	227±71 s	264±70 s	228±65 s	237±62 s	204±44 s
vs. 6 pm (%)				-0.4%	-6.8%	-2.3%	-6.7%	-12.3%$^+$	-16.2%*
Numeric	9	238±102 s	219±122 s	206±78 s	196±100 s	214±107 s	218±78 s	234±81 s	231±105 s
vs. 6 pm (%)				-13.2%	-10.6%	-9.8%	-0.8%	-1.4%	5.3%

Karolinska Sleepiness Scale (KSS)																
	6 pm				0 am				2 am				4 am			
	Placebo		Creatine		Placebo		Creatine		Placebo		Creatine		Placebo		Creatine	
	bef.	aft.	bef.	aft	bef.	aft.	bef.	aft.	bef.	aft.	bef.	aft.	bef.	aft.	bef.	aft.
**Score**	2.5	2.5	2.6	2.8	4.1	4.3	4.0	4.2	5.4	5.7	5.2	5.9	6.8	6.9	6.2	6.6
aft vs. bef	-0.1		0.3		0.2		0.3		0.3		0.7		0.1		0.4v	
vs. 6 pm (%)					68%**		8%		123% [c]		43%*		174% [f]		65%**	

Inverted Samn & Perelli Fatigue score (FAT)																
	6 pm				11 pm				2 am				4 am			
	Placebo		Creatine		Placebo		Creatine		Placebo		Creatine		Placebo		Creatine	
	bef	aft.	bef.	aft.	bef.	aft.	bef.	aft	bef.	aft.	bef.	aft	bef.	aft.	bef.	aft.
**Score**	6.6	7.8	5.7	6.4	9.0	9.7	8.2	8.5	10.7	12.4	10.0	11.7	13.4	13.8	12.5	13.6
aft vs. bef	1.1		0.8		0.7		0.3		1.6		1.7		0.4		1.2	
vs. 6 pm (%)					37%*		43%*		68%***		93%***		98%[b]		136%[c]	

$^+$ p= values of 0.0063, ≤ p ≤ 0.05, that did not survive Bonferroni correction, * p= values of p ≤ 0.0064, that survived Bonferroni correction, ** p= values of p ≤ 0.0005, *** p= values of p ≤ 0.00005.

Table S5
Outcome of cognitive tasks and scales for female participants. Mean number of correct results, processing time and intra-individual percentual changes of creatine versus placebo. Changes in Karolinska Sleepiness Scale (KSS) and Fatigue scale are shown below.

Creatine versus placebo					
**Task**	**No. of trials**	**Number of correct results (Score) vs. 6pm (%)**			
		0 am	2 am	4 am	all 3 time points pooled
WMT	22	4.0% ±4.7%	2.5% ±4.6%	0.7% ±6.4%	2.4% ±3.1%
Digit span	12	-26.2% ±18.5%	-3.3% ±22.7%	1.1% ±13.9%	-9.6% ±10.8%
N-back	42	3.3% ±4.2%	10.3% ±8.7%	-0.3% ±6.7%	4.4%± 4.0%
Language	21	9.8% ±4.0% $^{+}$	4.6% ±5.3%	0.3% ±0.3%	4.9% ±2.7%
Logic	17	10.5% ±4.0%$^{+}$	11.2% ±3.6%$^{+}$	15.1% ±6.4%$^{+}$	12.3% ±2.8%**
Numeric	9	4.1% ±5.9%	0.7% ±6.1%	10.1% ±6.0%	5.0% ±3.5%
**Task**	**No. of trials**	**Processing Time (t) vs. 6pm (%)**			
		0 am	2 am	4 am	all 3 time points pooled
WMT	22	-8.9% ±6.3%	-2.2%± 6.6%	1.9% ±14.0%	-3.1% ±5.6%
Digit SPAN	12	-25.9% ±21.9%	0.2%± 12.5%	-8.0% ±24.6%	-11.5% ±11.9%
Language	21	-17.3% ±11.3%	-8.9% ±11.4%	-28.5% ±12.5% $^{+}$	-18.2% ±2.7%$^{+}$
Logic	17	-6.9% ±6.3%	-11.1% ±9.5%	-12.7% ± 8.1%	-10.3% ±1.2%$^{+}$
Numeric	9	4.4% ±8.1%	-5.6% ±12.1%	-6.9% ±10.0%	-2.7% ±5.9%
**Karolinska Sleepiness Scale (KSS)**					
**Score**		0 am	2 am	4 am	all 3 time points pooled
**vs. 6 pm (%)**		-2.3±-0.3%	-7.8±-5.3%	6.8±-5.4%	-1.1±-3.0%
**Inverted Samn & Perelli Fatigue score (FAT)**					
**Score**		0 am	2 am	4 am	all 3 time points pooled
**vs. 6 pm (%)**		-7.9 ±7.2%	-9.9 ±12.6%	-17.8 ±25.7%	-11.9±8.2%

$^{+}$ p= values of 0.0063, ≤ *p* ≤ 0.05, that did not survive Bonferroni correction, * p= values of *p* ≤ 0.0064, that survived Bonferroni correction, ** p= values of *p* ≤ 0.0005, that survived Bonferroni correction, *** p= values of *p* ≤ 0.00005, that survived Bonferroni correction

Table S6

Outcome of cognitive tasks and scales for male participants. Mean number of correct results, processing time and intra-individual percentual changes of creatine versus placebo. Changes in Karolinska Sleepiness Scale (KSS) and Fatigue scale are shown below.

**Creatine versus placebo**					
**Task**	**No. of trials**	**Number of correct results (Score) vs. 6pm (%)**			
		0 am	2 am	4 am	all 3 time points pooled
WMT	22	-17.8%± 10.3%	-14.1%± 13.4%	0.8%± 10.7%	-10.4%± 6.8%
Digit span	12	-10.0% ± 13.4%	-19.7% ±36.0%	18.8% ±17.8%	-3.6% ± 14.4%
N-back	42	4.5%±13.1%	0.8% ±7.5%	13.7% ±10.6%	6.3% ± 6.2%
Language	21	-1.4%± 9.7%	-7.8%± 9.9%	-4.1% ±0.6%	-4.4% ±5.5%
Logic	17	-4.2%± 4.0%	-2.3% ±4.8%	-1.8% ±4.0%	-2.8% ±2.5%
Numeric	9	15.7% ±10.9%	4.9%± 7.1%	2.3%± 7.0%	7.6%± 5.9%
**Task**	**No. of trials**	**Processing Time (t) vs. 6pm (%)**			
		0 am	2 am	4 am	all 3 time points pooled
WMT	22	-7.9% ±6.5%	13.6% ±7.4%	16.4% ±14.7%	7.4% ±6.2%
Digit SPAN	12	-5.3% ±10.5%	6.7% ±17.7%	18.0% ±19.9%	6.5% ±9.7%
Language	21	-24.0% ±-14.5%	10.8% ±18.0%	1.2% ±16.4%	-4.0% ±9.8%
Logic	17	-3.9% ± 7.8%	9.7% ±9.4%	11.7% ±9.8%	5.8% ±5.4%
Numeric	9	-5.7% ±11.6%	10.8% ±12.3%	11.4% ±8.9%	5.5% ±6.5%
**Karolinska Sleepiness Scale (KSS)**					
**Score**		0 am	2 am	4 am	all 3 time points pooled
**vs. 6 pm (%)**		-10.8±4.5%	-0.7±0.6%	-2.7±1.9%	-4.7±1.7%
**Inverted Samn & Perelli Fatigue score (FAT)**					
**Score**		0 am	2 am	4 am	all 3 time points pooled
**vs. 6 pm (%)**		-27.3±2.9%	-2.1±0.4%	39.8±14.5%	3.5±-6.8%

[+] p= values of 0.0063, ≤ *p* ≤ 0.05, that did not survive Bonferroni correction, * p= values of *p* ≤ 0.0064, that survived Bonferroni correction, ** p= values of *p* ≤ 0.0005, that survived Bonferroni correction, *** p= values of *p* ≤ 0.00005, that survived Bonferroni correction

Table S7

Outcome of cognitive tasks and scales for vegetarian participants. Mean number of correct results, processing time and intra-individual percentual changes of creatine versus placebo. Changes in Karolinska Sleepiness Scale (KSS) and Fatigue scale are shown below.

**Creatine versus placebo**					
**Task**	**No. of trials**	**Number of correct results (Score) vs. 6pm (%)**			
		0 am	2 am	4 am	all 3 time points pooled
WMT	22	2.6% ±6.5%	10.0% ±6.5%	0.7% ±6.3%	4.4% ±3.8%
Digit span	12	-5.2% ± 15.8%	34.0% ±30.2%	-26.4% ± 22.6%	0.8% ±14.5%
N-back	42	11.6% ±3.7%[+]	18.0% ±8.8%	2.8% ±8.2%	10.8% ±4.4% [+]
Language	21	9.3% ±8.1%	5.1% ±9.1%	1.1% ±0.5%	5.3% ±4.3%
Logic	17	3.3% ±5.3%	6.0% ±6.1%	9.2% ±10.1%	6.2% ±4.3%
Numeric	9	-4.1% ±9.7%	-3.4% ±8.9%	4.1% ±8.6%	-1.1% ±5.3%
**Task**	**No. of trials**	**Processing Time (t) vs. 6pm (%)**			
		0 am	2 am	4 am	all 3 time points pooled
WMT	22	0.4% ±7.7%	11.6% ±6.2%	2.2% ±5.4%	4.7% ±3.8%
Digit SPAN	12	6.9% ±13.9%	19.4% ±15.2%	23.3% ±16.1%	16.5% ±8.8%
Language	21	-36.7% ±19.2%	-21.9% ±19.3%	-36.3% ±18.6%	-31.6% ± 11.1% [+]
Logic	17	-5.5% ±8.1%	-1.0% ±16.8%	-2.0% ±12.9%	-2.8% ±7.6%
Numeric	9	-1.9% ±10.6%	1.2% ±18.6%	-3.3% ±17.8%	-1.3% ±9.3%
**Karolinska Sleepiness Scale (KSS)**					
**Score**		0 am	2 am	4 am	all 3 time points pooled
**vs. 6 pm (%)**		-6.8±0.6%	-39.6±5.2%	-1.4±0.4%	-16.0±5.5%
**Inverted Samn & Perelli Fatigue score (FAT)**					
**Score**		0 am	2 am	4 am	all 3 time points pooled
**vs. 6 pm (%)**		-32.1±-12.7%	-21.2±-4.7%	29.9±-13.9%	-7.8±11.0%

[+] p= values of 0.0063, ≤ *p* ≤ 0.05, that did not survive Bonferroni correction, * p= values of *p* ≤ 0.0064, that survived Bonferroni correction, ** p= values of *p* ≤ 0.0005, that survived Bonferroni correction, *** p= values of *p* ≤ 0.00005, that survived Bonferroni correction

Table S8

Outcome of cognitive tasks and scales for non- vegetarian participants. Mean number of correct results, processing time and intra-individual percentual changes of creatine versus placebo. Changes in Karolinska Sleepiness Scale (KSS) and Fatigue scale are shown below.

Creatine versus placebo					
**Task**	**No. of trials**	**Number of correct results (Score) vs. 6pm (%)**			
		0 am	2 am	4 am	all 3 time points pooled
WMT	22	-8.5% ±7.2%	-10.8% ±8.3%	0.8% ±7.9%	-6.2% ±4.6%
Digit span	12	-25.9% ±15.7%	-31.4% ±24.6%	24.1% ±10.8%	-10.7% ±10.8%
N-back	42	3.4% ±8.3%	1.2%± 7.6%	6.7% ±7.9%	3.7% ±4.6%
Language	21	0.2% ±6.2%	-3.1%± 6.4%	-2.7% ±0.4%	-1.9% ±3.5%
Logic	17	4.9% ±3.9%	5.5% ±3.7%	7.6% ±4.4%	6.0% ±2.3%$^{+}$
Numeric	9	8.9% ±5.8%	2.4% ±4.6%	6.9% ±4.6%	6.1% ±2.9%$^{+}$
**Task**	**No. of trials**	**Processing Time (t) vs. 6pm (%)**			
		0 am	2 am	4 am	all 3 time points pooled
WMT	22	-12.5% ±5.4%$^{+}$	1.1% ± 6.8%	10.4% ± 14.7%	-0.3% ±5.8%
Digit SPAN	12	-28.3% ±18.3%	-4.8% ± 13.2%	-14.4% ±22.8%	-16.0% ±10.8%
Language	21	-12.6%±-9.3%	8.7% ± 11.3%	-7.2% ± 11.9%	-3.7% ± 6.4%
Logic	17	-5.7% ± 6.1%	-3.3% ± 6.9%	-2.9% ± 7.7%	-4.0% ± 4.0%
Numeric	9	0.2% ±6.8%	1.2% ±8.9%	0.7% ±7.2%	0.7% ±4.4%
**Karolinska Sleepiness Scale (KSS)**					
**Score**		0 am	2 am	4 am	all 3 time points pooled
**vs. 6 pm (%)**		-5.4±2.6%	10.8±7.4%	4.8±3.4%	3.4±1.2%
**Inverted Samn & Perelli Fatigue score (FAT)**					
**Score**		0 am	2 am	4 am	all 3 time points pooled
**vs. 6 pm (%)**		-8.6±7.5%	-0.1±0.1%	-4.7±6.2%	-4.5±2.8%

$^{+}$ p= values of 0.0063, ≤ *p* ≤ 0.05, that did not survive Bonferroni correction, * p= values of *p* ≤ 0.0064, that survived Bonferroni correction, ** p= values of *p* ≤ 0.0005, that survived Bonferroni correction, *** p= values of *p* ≤ 0.00005, that survived Bonferroni correction

Table S9. Results in psychomotor vigilance tests (PVT) of reaction time (TR) and reaction time distribution (RTD) in the placebo and creatine session for female participants. The tests lasted 8 min. Trials with reaction times ≥ 1000ms were considered as lapses.

PVT (Placebo)

	RTD	Mean RT	Median RT	Minimum RT	Maximum RT	Fastest 10%	Slowest 10%	Speed
6pm	40±8 ms	284±23 ms	273±21 ms	202±13 ms	488±76 ms	217±14 ms	398±52 ms	4±0.3 1/s
0 am	44±7 ms	296±29 ms	287±30 ms	203±31 ms	557±203 ms	227±22 ms	417±45 ms	4±0.4 1/s
vers. 6pm	9.5±3%	4.3±2%[+]	5.0±2%[+]	0.5±3%	14±11%	5±2%[+]	5±3%	-4±1%[+]
2 am	44±8 ms	307±34 ms	293±32 ms	211±19 ms	601±250 ms	229±19 ms	454±82 ms	3±0.4 1/s
vers. 6pm	11.1±5%[+]	8.2±2%*	7.4±2%*	4.8±2%[+]	23.1±12%	5.4±2%[+]	14.0±4%[+]	-6.3±1%*
4apm	43±7 ms	311±33 ms	294±33 ms	209±13 ms	601±218 ms	228±20 ms	472±67 ms	3±0.3 1/s
vers. 6pm	10.2±4%	9.7±2%**	7.6±2%*	3.8±1%	23.2±4%	4.9±1%*	18.5±3%**	-7.0±2%**
vers. 6pm Pooled	10.3±2%***	7.4±1.1%[c]	6.6±1.2% [b]	3.0±1.3%[+]	20.1±6.5%*	5.0±0.9% [a]	12.4±2.3% [a]	-5.7±0.9% [b]

PVT (Creatine)

	RTD	Mean RT	Median RT	Minimum RT	Maximum RT	Fastest 10%	Slowest 10%	Speed
6pm	40±8 ms	282±15 ms	273±14 ms	205±15 ms	483±77 ms	219±15 ms	395±28 ms	3.7±0.21/s
0 am	41±7 ms	290±29 ms	280±25 ms	208±15 ms	516±118 ms	221±16 ms	410±60 ms	3.6±0.3 1/s
vers. 6pm	2.4±6%	2.0±2%	2.7±2%	1.3±2%	6.9±7%	0.7±2%	3.9±3%	-1.7±2%
2 am	43±8 ms	294±23 ms	283±21 ms	207±17 ms	525±67 ms	225±17 ms	421±38 ms	3.5±0.3 1/s
vers. 6pm	5.7±5%	4.2±2%[+]	3.8±2%	1.1±2%	8.7±6%	2.7±2%	6.7±2%[+]	-3.4±2%
4apm	42±12 ms	299±36 ms	287±36 ms	208±24 ms	553±182 ms	226±23 ms	430±66 ms	3.5±0.4 1/s
vers. 6pm	5.1±5%	5.8±2%[+]	5.3±3%	1.5±2%	14.4±10%	3.1±2%	8.9±4%[+]	-3.9±2%
vers. 6pm Pooled	4.4±3.1%	4.2±1.2%*	3.9±1%*	1.3±1%	10.0±4%[+]	2.2±1%[+]	6.5±1%*	-3.0±1%[+]

PVT (Creatine vs. Placebo)

vers. 6pm	RTD	Mean RT	Median RT	Minimum RT	Maximum RT	Fastest 10%	Slowest 10%	Speed
0 am	-11.0±6.4%	-2.0±1.8%	-2.6±2.1%	0.5±3.5%	-11.9±16.3%[+]	-4.1±1.9%[+]	-1.5±4.4%	1.8±1.6%
2 am	-7.9±5.8%	-4.3±2.2%	-3.9±2.2%	-3.9±2.7%	-19.0±12.6%	-2.8±2.0%	-8.3±5.2%	2.6±1.9%
4am	-9.9±8.3%	-6.6±3.1%	-5.1±2.8%	-5.3±2.9%	-29.0±20%	-2.6±2.2%	-16.7±7.3%	4.5±2.4%
Pooled	-9.6±4.0%[+]	-4.3±1.4%[+]	-3.9±1.4%[+]	-2.9±1.8%	-20.0±9.6%	-3.2±1.2%[+]	-8.8±3.4%[+]	3.0±1.2%[+]

[+]p= values of 0.0063, ≤ p ≤ 0.05, that did not survive Bonferroni correction, * p= values of $p \leq 0.0064$, that survived Bonferroni correction, ** p= values of $p \leq 0.0005$, that survived Bonferroni correction, *** p= values of $p \leq 0.00005$, that survived Bonferroni correction, [a–e] p $\leq 5.0 \times 10^{-6}$ to 5.0×10^{-12} in decadal steps.

Table S10. Results in psychomotor vigilance tests (PVT) of reaction time (TR) and reaction time distribution (RTD) in the placebo and creatine session for male participants. The tests lasted 8 min. Trials with reaction times ≥ 1000ms were considered as lapses.

PVT (Placebo)								
	RTD	Mean RT	Median RT	Minimum RT	Maximum RT	Fastest 10%	Slowest 10%	Speed
6pm	35±17 ms	274±27 ms	262±25 ms	197±30 ms	466±74 ms	215±20 ms	382±51 ms	3.8±0.4 1/s
0 am	41±12 ms	286±37 ms	274±32 ms	203±22 ms	588±266 ms	221±24 ms	414±83 ms	3.7±0.5 1/s
vers. 6pm	17.4±9%	4.6±2%	4.3±2%	2.8±4%	26.2±15%	3.0±2%	8.5±5%	-3.5±2%
2 am	43±9 ms	301±42 ms	283±34 ms	207±19 ms	592±244 ms	226±20 ms	455±106 ms	3.5±0.4 1/s
vers. 6pm	23.1±9%	10.0±3%[+]	8.0±3%[+]	5.1±3%	27.1±16%	5.2±1%*	19.2±7%[+]	-7.4±2%*
4apm	45±8 ms	330±45 ms	307±37 ms	213±20 ms	861±443 ms	231±19 ms	562±149 ms	3.3±0.4 1/s
vers. 6pm	30.5±6%[+]	20.4±4%**	17.0±3%**	7.8±2%	84.8±12%[+]	7.6±1% **	47.2±13%*	-13.8±2%***
vers. 6pm Pooled	23.7±5%**	11.6±2%[a]	9.8±2% [a]	5.2±2%[+]	46.0±12%*	5.3±0.9% [a]	25.0±5%***	-8.2±1% [b]

PVT (Creatine)								
	RTD	Mean RT	Median RT	Minimum RT	Maximum RT	Fastest 10%	Slowest 10%	Speed
6pm	39±10 ms	278±28 ms	266±27 ms	192±28 ms	523±170 ms	215±19 ms	396±70 ms	3.7±0.4 ms
0 am	42±11 ms	305±52 ms	285±42 ms	201±37 ms	679±470 ms	228±25 ms	475±165 ms	3.5±0.5 1/s
vers. 6pm	7.5±5%	9.7±4%[+]	7.1±3%[+]	5.0±6%	29.7±24%	5.7±2%	19.7±10%	-6.2±2%[+]
2 am	43±8 ms	338±100 ms	306±56 ms	214±24 ms	799±552 ms	234±26 ms	603±432 ms	3.3±0.6 1/s
vers. 6pm	10.1±5%	21.3±9%[+]	14.8±4%[+]	11.8±5%[+]	52.6±30%	8.5±3%[+]	52.2±30%	-10.7±3%*
4apm	44±10 ms	336±80 ms	309±61	222±27	843±531	238±31	554±218	3.3±0.61/s
vers. 6pm	11.2±6%	20.8±7%[+]	16.0%±5%[+]	15.7%±5%[+]	61.0%±29%	10.7%±3%[+]	39.7%±14%[+]	-12.4%±3%*
vers. 6pm Pooled	9.6±3%	17.3±4%**	12.7±2%***	10.8±3%*	47.8±16%*	8.3±1%***	37.2±11%*	-9.8±1%[b]

PVT (Creatine vs. Placebo)								
**vers. 6pm**	RTD	Mean RT	Median RT	Minimum RT	Maximum RT	Fastest 10%	Slowest 10%	Speed
0 am	-3.1±8.0%	3.7±3.3%	2.3±3.7%	3.2±3.9%	-9.0±12.9%	3.1±3.1%	4.9±5.3%	-3.0±3.1%
2 am	-12.8±13.1%	4.0±3.9%	4.4±3.8%	5.5±5.4%	6.5±15.5%	2.7±3.3%	7.7±7.9%	-2.4±3.3%
4am	-10.6±11.2%	2.3±4.9%	1.5±4.8%	6.2±3.6%	-9.8±26.4%	2.6±2.5%	0.2±9.3%	-1.3±3.7%
Pooled	-8.8±6.4%	3.3±2.4%	2.7±2.4%	5.0±2.5%	-4.1±11.1%	2.8±1.7%	4.3±4.3%	-2.2±1.9%

[+]p= values of 0.0063, ≤ *p* ≤ 0.05, that did not survive Bonferroni correction, * p= values of *p* ≤ 0.0064, that survived Bonferroni correction, ** p= values of *p* ≤ 0.0005, that survived Bonferroni correction, *** p= values of *p* ≤ 0.00005, that survived Bonferroni correction, [a–c] p ≤ 5.0×10^{-6} to 5.0×10^{-10} in decadal steps.

Table S11. Results in psychomotor vigilance tests (PVT) of reaction time (TR) and reaction time distribution (RTD) in the placebo and creatine session for vegetarian participants. The tests lasted 8 min. Trials with reaction times ≥ 1000ms were considered as lapses.

PVT (Placebo)								
	**RTD**	**Mean RT**	**Median RT**	**Minimum RT**	**Maximum RT**	**Fastest 10%**	**Slowest 10%**	**Speed**
6pm	33±18 ms	274±28 ms	264±25 ms	199±14 ms	493±86 ms	214±17 ms	381±51 ms	4±0.4 1/s
0 am	40±10 ms	286±34 ms	277±32 ms	204±18 ms	511±148 ms	221±21 ms	406±69 ms	4±0.5 1/s
vers. 6pm	22.1±3%	4.5±2%	4.8±3%	2.7±2%	3.5±9%	3.5±2%	6.3±3%	-3.8±2%
2 am	44±10 ms	308±50 ms	294±44 ms	213±22 ms	570±158 ms	228±24 ms	461±111 ms	3.5±0.6 1/s
vers. 6pm	33.7±13%$^{+}$	12.5±4%$^{+}$	11.2±4%$^{+}$	7.4±2%$^{+}$	15.6±9%	6.9±2%$^{+}$	20.9±8%$^{+}$	-8.7±3%$^{+}$
4apm	43±8 ms	315±46 ms	298±41	211±15	615±246	229±19	488±128	3±0.51/s
vers. 6pm	33.5±6%$^{+}$	14.9±4%*	12.9±4%$^{+}$	6.4±1%$^{+}$	24.7±5%	7.4±2%*	27.9±5%$^{+}$	-10.6±3%*
vers. 6pm Pooled	29.8±7%**	10.6±2%***	9.6±2%**	5.5±1%**	14.6±6%$^{+}$	5.9±1%***	18.4±4%**	-7.7±1%***

PVT (Creatine)								
	**RTD**	**Mean RT**	**Median RT**	**Minimum RT**	**Maximum RT**	**Fastest 10%**	**Slowest 10%**	**Speed**
6pm	40±11 ms	278±24 ms	269±23 ms	207±16 ms	472±52 ms	220±19 ms	384±41 ms	3.7±0.3 1/s
0 am	40±10 ms	285±31 ms	275±32 ms	207±17 ms	501±67 ms	221±20 ms	398±42 ms	3.6±0.4 1/s
vers. 6pm	-0.3±8%	2.5±2%	2.2±3%	0.0±2%	6.2±6%	0.6±2%	3.5±3%	-1.6±2%
2 am	45±7 ms	300±40 ms	291±38 ms	210±23 ms	508±109 ms	229±21 ms	420±74 ms	3.5±0.5 1/s
vers. 6pm	11.1±8%	7.7±3%	8.04%±	1.3±4%	7.7±5%	4.1±3%	9.3±4%	-5.8±3%
4apm	45±14 ms	303±50 ms	290±44 ms	212±32 ms	550±222 ms	231±28 ms	433±107 ms	3.5±0.5 1/s
vers. 6pm	11.6±8%	9.0±4%	7.6±3%	2.2±4%	16.7±13%	5.1±3%	12.6±7%	-6.1±3%
vers. 6pm Pooled	7.3±5%	6.4±2%*	5.9±2%*	1.2±2%	10.2±5%	3.3±1%$^{+}$	8.5±3%$^{+}$	-4.5±2%$^{+}$

PVT (Creatine vs. Placebo)								
**vers. 6pm**	**RTD**	**Mean RT**	**Median RT**	**Minimum RT**	**Maximum RT**	**Fastest 10%**	**Slowest 10%**	**Speed**
0 am	-15.5±15%	-2.1±2.6%	-2.7±3.2%	-2.8±2.1%	1.2±13.8%	-2.9±2.3%	-2.8±4.5%	2.1±2.4%
2 am	-24.8±14.7%	-4.8±3.2%	-2.9±3%	-5.9±3.7%	-8.7±7.1%	-2.8±2.4%	-11.7±6.7%	2.9±2.7%
4am	-9.3±15.3%	-6.3±5.7%	-5.2±4.9%	-3.6±4.5%	-15.9±20.8%	-0.1±3.8%	-17.5±11%	5.8±3.7%
vers. 6pm Pooled	-16.5±8%	-4.4±2.4%	-3.6±2.2%	-4.1±2.1%	-7.8±8.8%	-1.9±1.7%	-10.6±4.7%$^{+}$	3.6±1.7%

$^{+}$ p= values of 0.0063, ≤ *p* ≤ 0.05, that did not survive Bonferroni correction, * p= values of *p* ≤ 0.0064, that survived Bonferroni correction, ** p= values of *p* ≤ 0.0005, *** p= values of *p* ≤ 0.00005.

Table S12 Results in psychomotor vigilance tests (PVT) of reaction time (TR) and reaction time distribution (RTD) in the placebo and creatine session for non-vegetarian participants. The tests lasted 8 min. Trials with reaction times ≥ 1000ms were considered as lapses.

PVT (Placebo)								
	**RTD**	**Mean RT**	**Median RT**	**Minimum RT**	**Maximum RT**	**Fastest 10%**	**Slowest 10%**	**Speed**
6pm	40±8 ms	283±24 ms	271±22 ms	201±25 ms	473±70 ms	218±16 ms	396±52 ms	3.7±0.31/s
0 am	44±10 ms	295±32 ms	284±31	203±30 ms	596±257 ms	227±23 ms	420±61 ms	3.5±0.41/s
vers. 6pm	9.0±3%	4.4±2%[+]	4.7±2%[+]	0.9±3%	26.2±13%	4.2±1%[+]	6.2±4%	-3.6±1%[+]
2 am	43±7 ms	303±30 ms	287±27 ms	208±17 ms	610±278 ms	228±17 ms	451±83 ms	3.5±0.31/s
vers. 6pm	9.0±2%[+]	7.4±2%**	6.0±2%*	3.8±2%	29.0±13%[+]	4.6±1%*	14.0±4%[+]	-5.9±1%*
4apm	45±8 ms	321±36 ms	300±32 ms	211±17 ms	751±386 ms	229±19 ms	519±112 ms	3.3±0.3 1/s
vers. 6pm	12.3±4%*	13.7±3%***	10.7±2%**	5.0±2%[+]	59.0±23%[+]	5.4±1%*	31.1±8%**	-9.6±2%***
vers. 6pm Pooled	10.2±2%[a]	8.5±1% [d]	7.2±1% [c]	3.2±1%[+]	38.1±9%***	4.7±1% [b]	17.1±3% [a]	-6.3±2% [d]
**PVT (Creatine)**								
	**RTD**	**Mean RT**	**Median RT**	**Minimum RT**	**Maximum RT**	**Fastest 10%**	**Slowest 10%**	**Speed**
6pm	40±9 ms	282±21 ms	271±20 ms	196±25 ms	513±146 ms	217±16 ms	401±53 ms	3.7±0.3 1/s
0 am	42±10 ms	301±44 ms	286±34 ms	205±30 ms	621±384 ms	225±21 ms	455±139 ms	3.5±0.4 1/s
vers. 6pm	6.7±3%	6.8±4%[+]	5.6±2%[+]	4.1±4%	21.2±16%	3.7±2%[+]	13.4±7%	-4.5±2%[+]
2 am	42±8 ms	318±80 ms	294±43 ms	211±20 ms	697±444 ms	229±23 ms	532±344 ms	3.4±0.4 1/s
vers. 6pm	6.0±4%	12.8±6%[+]	8.4±3%[+]	7.3±3%[+]	36.1±19%	5.5±2%[+]	32.5±18%	-6.8±2%*
4apm	42±10 ms	320±65 ms	300±51 ms	215±24 ms	728±442 ms	232±27 ms	504±176 ms	3.4±0.5 1/s
vers. 6pm	5.7±5%	13.2±4%[+]	10.6±4%[+]	9.5±3%[+]	42.1±19%[+]	6.7±2%[+]	25.5±9%[+]	-8.1±2%*
vers. 6pm Pooled	6.1±2%[+]	10.9±3%**	8.2±2%***	7.0±2%*	33.1±11%*	5.3±1%**	23.8±7%*	-6.5±1%[a]

PVT (Creatine vs. Placebo)								
**vers. 6pm**	**RTD**	**Mean RT**	**Median RT**	**Minimum RT**	**Maximum RT**	**Fastest 10%**	**Slowest 10%**	**Speed**
0 am	-4.1±5.2%	1.3±2.2%	0.3±2.4%	3.5±3.5%	-15.5±14.1%	-0.5±2.3%	2.7±4.3%	-1.1±1.9%
2 am	-2.7±5.3%	0.7±2.7%	0.5±2.8%	2.5±3.6%	-9.1±14.4%	0.3±2.4%	2.5±5.6%	-0.4±2.3%
4am	-10.5±6.7%	-1.7±3.1%	-1.2±3.1%	0.5±2.9%	-24.1±21%	-0.8±1.8%	-6.6±6.9%	0.6±2.5%
Pooled	-5.8±3.4%	0.1±1.6%	-0.2-±1.6%	2.2±1.9%	-16.2±9.8%	-0.3±1.3%	-0.4±3.3%	-0.3±1.3%

[+]p= values of 0.0063, $\leq p \leq 0.05$, that did not survive Bonferroni correction, * p= values of $p \leq 0.0064$, that survived Bonferroni correction, ** p= values of $p \leq 0.0005$, that survived Bonferroni correction, *** p= values of $p \leq 0.00005$, that survived Bonferroni correction, [a–d] p $\leq 5.0 \times 10^{-6}$ to 5.0×10^{-9} in decadal steps.

Table S13 Pearson's correlation coefficients (r_p, p-value) between changes in FAT, KSS score and cognitive score in females and males after creatine or placebo administration when pooled at all 3 timepoints (0pm, 2am, 4a).

Placebo (Female)							
	PVT (RT)	WMT	SPAN	N-Back	Language	Logic	Numeric
FAT	0.27	0.10	0.22	-0.01	0.04	-0.01	-0.08
KSS	0.23	-0.12	0.03	-0.16	-0.08	0.05	-0.11
Placebo (Female)							
	PVT (RT)	WMT	SPAN	N-Back	Language	Logic	Numeric
FAT	0.13	0.02	-0.02	-0.20	-0.33	-0.12	-0.31
KSS	0.19	0.12	-0.02	-0.10	-0.27	-0.03	-0.20
Placebo (Male)							
	PVT (RT)	WMT	SPAN	N-Back	Language	Logic	Numeric
FAT	0.33	-0.27	-0.01	-0.33	-0.28	0.18	0.20
KSS	0.67***	-0.26	-0.19	-0.04	-0.07	0.03	0.18
Creatine (Male)							
	PVT (RT)	WMT	SPAN	N-Back	Language	Logic	Numeric
FAT	0.24	-0.31	0.05	0.14	0.12	-0.14	-0.30
KSS	0.30	-0.59**	0.21	0.17	-0.07	-0.15	-0.20

** p= values of $p \leq 0.0005$, *** p= values of $p \leq 0.00005$

Table S14 Pearson's correlation coefficients (r_p, p-value) between changes in FAT und KSS score and cognitive score in vegetarians and non-vegetarians after creatine or placebo administration when pooled at all 3 timepoints (0pm, 2am, 4a).

Placebo (Vegetarian)							
	PVT (RT)	WMT	SPAN	N-Back	Language	Logic	Numeric
FAT	0.35	0.01	-0.07	0.28	-0.09	0.07	0.15
KSS	0.34	0.03	-0.16	0.14	-0.08	0.01	0.08
**Creatine (Vegetarian)**							
	PVT (RT)	WMT	SPAN	N-Back	Language	Logic	Numeric
FAT	0.24	-0.31	0.05	0.22	0.12	-0.14	-0.30
KSS	0.30	-0.59**	0.21	0.51	-0.07	-0.15	-0.20
**Placebo (non-Vegetarian)**							
	PVT (RT)	WMT	SPAN	N-Back	Language	Logic	Numeric
FAT	0.19	-0.03	0.26	-0.23	-0.04	-0.01	-0.07
KSS	0.47**	-0.27	-0.01	-0.18	-0.03	0.05	-0.03
**Creatine (non-Vegetarian)**							
	PVT (RT)	WMT	SPAN	N-Back	Language	Logic	Numeric
FAT	0.03	-0.22	-0.06	-0.07	-0.01	-0.29	-0.42*
KSS	0.19	-0.36*	0.07	0.23	-0.12	-0.24	-0.26

+ p= values of 0.0063, ≤ p ≤ 0.05, that did not survive Bonferroni correction, * p= values of p ≤ 0.005, ** p= values of p ≤ 0.0005
